# Comprehensive snapshots of natural killer cells functions, signaling, molecular mechanisms and clinical utilization

**DOI:** 10.1038/s41392-024-02005-w

**Published:** 2024-11-08

**Authors:** Sumei Chen, Haitao Zhu, Youssef Jounaidi

**Affiliations:** 1 Department of Radiation Oncology, Hangzhou Cancer Hospital, Hangzhou, Zhejiang China; 2 Department of Hepatobiliary Surgery, The Affiliated Hospital of Guizhou Medical University, Guiyang, Guizhou China; 3 Department of Anesthesia, Critical Care and Pain Medicine, Massachusetts General Hospital and Harvard Medical School, Boston, MA USA

**Keywords:** Innate immunity, Cancer microenvironment, Immunotherapy

## Abstract

Natural killer (NK) cells, initially identified for their rapid virus-infected and leukemia cell killing and tumor destruction, are pivotal in immunity. They exhibit multifaceted roles in cancer, viral infections, autoimmunity, pregnancy, wound healing, and more. Derived from a common lymphoid progenitor, they lack CD3, B-cell, or T-cell receptors but wield high cytotoxicity via perforin and granzymes. NK cells orchestrate immune responses, secreting inflammatory IFNγ or immunosuppressive TGFβ and IL-10. CD56^dim^ and CD56^bright^ NK cells execute cytotoxicity, while CD56^bright^ cells also regulate immunity. However, beyond the CD56 dichotomy, detailed phenotypic diversity reveals many functional subsets that may not be optimal for cancer immunotherapy. In this review, we provide comprehensive and detailed snapshots of NK cells’ functions and states of activation and inhibitions in cancer, autoimmunity, angiogenesis, wound healing, pregnancy and fertility, aging, and senescence mediated by complex signaling and ligand-receptor interactions, including the impact of the environment. As the use of engineered NK cells for cancer immunotherapy accelerates, often in the footsteps of T-cell-derived engineering, we examine the interactions of NK cells with other immune effectors and relevant signaling and the limitations in the tumor microenvironment, intending to understand how to enhance their cytolytic activities specifically for cancer immunotherapy.

## Introduction

Natural killer (NK) cells were first described as killer lymphocytes that induce rapid leukemia cell death without requiring soluble factors^1^ and separately, in the same year as lymphocytes distinct from T-cells but capable of killing tumors caused by viruses.^2^ The knowledge accumulated since then has revealed the complexity of NK cell biology and interactions with cancer cells and virus-infected cells. It also revealed their role in autoimmunity,^3–5^ angiogenesis,^6^ wound healing,^7–9^ pregnancy and fertility,^10^ aging,^11^ disease, and senescence.^12,13^ NK cells are large granular lymphocytes sharing a common lymphoid progenitor with two pillars of adaptive immunity: lymphocytes B and T-cells. However, NK cells do not express CD3, B-cell receptor, or T-cell receptor. A defining feature of NK cells is their high cytotoxicity, rapid recognition, and elimination of threats, suggesting a strong evolutionary pressure in organisms without adaptive immunity to have fast-acting and efficient NK cells with an adequate array of activating receptors to survive insults such as viral infections and intrusion by non-self. NK cells are unique among innate immune cells since they use tools similar to adaptive immunity to resolve these insults. Eliminating these cells by NK cells is achieved, as in the case of T-Cells, by using pore-forming perforin^14^ designed to create pores with an inner diameter of ~16 nm^15^ in the target cell membrane and delivery of proteolytic granzymes^16^ that activate Caspase-3 and 10 to trigger apoptosis and Granulysin (GNLY). This saponin-like toxin lyses bacteria such as Mycobacterium tuberculosis,^17^ preventing intracellular bacteria’s escape.^18^ During pregnancy, decidual NK (dNK) cells can deliver GNLY via nanotubes to surgically kill bacteria inside the infected trophoblast without harming it.^19^ Similar delivery of GLNY is also performed by peripheral blood (PB) NK cells in infected macrophages and dendritic cells (DCs). NK cell’s cellular granularity is due to cytoplasmic vesicles filled with perforin and several granzymes. These granules and the Golgi apparatus all become polarized toward the point of contact with the targeted cell, called synapse, where the cargo is concentrated and delivered.^20,21^ However, despite this arsenal, NK cells may not eliminate large tumors or systemic viral infections. Their role appears to have been defined by evolution as first responders to deal with emerging threats in collaboration with other components of innate immunity, such as macrophages until adaptive immunity is fully deployed. NK cells are at the center of innate immunity with a presence in strategic organs that constitute barriers, such as the skin, gut, lungs, liver, uterus, breasts, and blood, where NK cells represent 5–15% of the lymphocyte population. In these organs, NK cells could play either an inflammatory role or, counterintuitively, an immunosuppressive one. In the first scenario, they increase inflammation after activation by tumor and virus-infected cells by secreting inflammatory cytokines such as INFγ,^22,23^ which activates macrophages,^24^ T-cells,^25^ and B-cells.^26^ However, cancer cells treated with IFNγ become resistant to NK cells, suggesting that NK secretion of IFNγ is also designed to involve other immune cells.^27^ NK cells are also the only lymphocytes that constitutively secrete TGFβ^28^ to reduce inflammation and inhibit T-cells cytotoxicity and proliferation,^29^ allowing tissue repair.^30^ Additionally, there is an increased frequency of autocrine TGFβ signaling by TGFβ-producing NK cells in patients with breast cancer.^31^ NK cells can also secrete immunosuppressive IL-10 in an early response to systemic, but not local infection.^32,33^ This secreted IL-10 indirectly limits T-cell activation by blocking APCs secretion of IL-12 and producing factors involved in antigen presentation^34^ and T-cell anti-viral response,^35^ thus promoting T-cell exhaustion^36^ and reducing immune-mediated damage to the host. IL-10, however, improves the effector functions and metabolism of NK cells via the mTOR pathway.^37^ Therefore, NK cells also have an immunomodulatory role and can influence innate and adaptive immunity through these anti- and pro-inflammatory roles.

The expression level of NK marker CD56 commonly defines the oversimplistic distinction between NK cells mediating these two functions. CD56^dim^ NK cells are efficient killers and produce more perforin and granzymes, while CD56^bright^ NK cells, which produce INFγ, TNFβ, IL-10, IL-13, and GM-CSF, also have immunomodulatory and suppressive functions.^38–40^ A new refinement of this classification has recently delineated three major populations of NK cells in PB.^41,42^ However, mass cytometry analysis considering twenty-eight NK cell receptors revealed an astounding 6000 to 30,000 phenotypic populations within an individual, where inhibitory receptors are determined by genetics and activating receptors are by the environment.^43^ Most circulating NK cells, ~90%, are CD56^dim,^ suggesting that circulating NK cells primary function is to eliminate rapidly targeted cells. Most CD56^dim^ cell subset also expresses CD16 (FcγRIII, Fc gamma receptor III),^40^ which is necessary for ADCC, again bridging innate and adaptive immunity.

## A brief history of five decades of progress in natural killer cell research

**Fig. 1:**
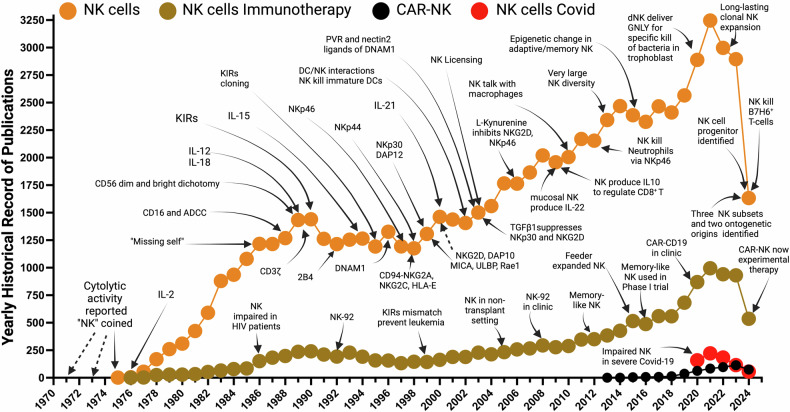
Historical narrative of important milestones in NK cell research. Interrogation of the historical record of natural killer cell research from PubMed using the keywords “Natural Killer cells”, “Natural Killer cells and Immunotherapy”, “Natural Killer cells and CAR-NK”, and “Natural Killer cells and Covid”. We provide in the main text of the review a year-by-year narrative of the progress/discovery culminating in the offering of CAR-NK as an “experimental therapy” against cancer at MD Anderson cancer center. In 2021, the number of publications related to “NK cells” is 3.57-fold less than “T-Cells” and the research record of “CAR-NK” is even more minuscule. Both fields show a subsequent slump in research publications in the period 2020–2023 which may be due to the Covid-19 pandemic

In 1971, even before NK cells formal identification, radioresistant lymphoid cells in lethally irradiated mice were reported to reject allogenic bone marrow,^44^ and the cytolytic activity attributed to PB lymphocytes was reported in 1973.^45^ In 1975, the term NK “Natural Killer” was coined^1^ (Fig. 1), and the discovery of IL-2 the same year, later revolutionized NK cell studies.^46^ In 1986, the “Missing-self” hypothesis was advanced to explain how NK cells pull the trigger.^47^ Also, in 1986, impaired activity of NK cells in HIV patients was reported.^48^ In 1988, NK cells were found to express CD16 and to mediate ADCC.^49^ In 1989, two CD56 subsets (dim and bright) were identified,^50^ and “interferon-inducing” IL-12^51^ and IL-18, crucial for NK activity, were discovered. Also, in 1989, the CD3ζ chain was discovered^52^ and shown to transduce CD16 signaling.^53^ In 1990, surface antigens with a role in cell activation and regulation of cytolytic function (later called KIRs) in NK cells were reported.^54^ In 1992, the first NK cell-activating receptor, 2B4, was discovered.^55^ The “Missing-self” hypothesis implied the existence of inhibitory receptors such as Ly49,^56^ first found in 1992 in mice, then in 1995 in humans, the KIRs^57–59^ that bind to MHC I were cloned/identified. A year earlier (1994), Klingemann published the NK cell line NK-92,^60^ established in 1992 and later used as a model in many NK studies. Cytokine IL-15, necessary for NK cell development, was also discovered in 1994.^61,62^ In 1996 NK cell activator DNAM-1 was discovered, first in T-cells.^63^ Natural cytotoxicity receptors (NCRs) will be discovered in succession: NKp46^64^ in 1997, NKp44^65^ in 1998, and in 1999, NKp30^66^ and adapter DAP12.^67^ In 1998 the inhibitory NKG2A and activating NKG2C receptors interactions with HLA-E were identified.^68^ In 1999, NKG2D receptor and adapter DAP10 activation by MICA^69^ and later in 2000 with ULBP^70^ and Retinoic acid early inducible gene (Rae1)^71^ were reported. Also, in 2000, IL-21 was discovered and found to expand NK cells.^72^ In 1999, the role of NK cells emerged in lowering the rates of leukemia relapse in MHC class I and KIR mismatch between the donor and recipient of hematopoietic stem cell transplants in a transplant setting.^73^ In 2002, the interactions between NK and DC cells were discovered.^74–76^ In 2003, TGFβ1 was found to impact the interaction between DCs and NK cells by suppressing NKp30 and NKG2D.^77^ In the same year, PVR and Nectin 2 were identified as ligands for DNAM-1.^78^ In 2005, Miller et al. pioneered the first use of NK cells in a non-transplant setting and showed the benefit of lymphocyte depletion preconditioning on NK cell expansion and persistence in vivo.^79^ In 2006, a component of the TME, Tryptophan metabolite, L-Kynurenine was reported to inhibit surface expression of NKp46 and NKG2D.^80^ In 2008, NK-92’s first phase I clinical trial was published.^81^ In 2009, NK cell secretion of IL-10 was reported to regulate CD8^+^T cells to prevent damage^35^ and another mucosal NK cell subset was found to produce IL-22.^82^ In 2010, NK cell interaction with macrophages was identified,^83^ and later in 2012, NK cells were reported to kill Neutrophils.^84^ In 2012, memory-like human NK cells that expand after transplantation are described.^85^ In 2015, evidence of adaptive or memory NK cells emerged after epigenetic changes (hypermethylation of Syk gene promoter) were found in NK cells in response to CMV infection.^86,87^ Also, in 2015, the first clinical trial using feeder-expanded NK cells showed safety and efficacy.^88^ In 2016, cytokine-induced memory-like NK cells were used in a phase-I clinical trial to show safety and efficacy.^89^ In 2020, CAR-NK (CD19) cells were used in a landmark clinical study to show safety and efficacy.^90^ In 2020, severely impaired NK cells were found in severe COVID-19 patients, and these NK cells were unable to kill overactive and inflammatory macrophages.^91^ Also in 2020, NK cells were discovered to specifically deliver Ganulysin, via nanotubes, to bacteria-infected trophoblasts, DCs and macrophages, without harm.^19^ In 2022, long-lasting NK cell clonal expansion from HCMV^+^ patients was reported.^92^ In early 2024, three major populations of NK cells are identified in PB.^41,42^ In mid-2024, CAR-NK cells are offered as an experimental option for cancer treatment at MD Anderson, and two NK cell lineage progenitors are identified in two seminal papers.^93,94^ Also, by mid-2024, NK cells were reported to kill, via NKp30, activated T-cells and CAR-CD19 T-cells expressing B7H6.^95^

## NK interaction with the major histocompatibility complex class I molecules

A critical regulator of NK cell reactivity is the major histocompatibility complex (MHC I). NK and T-cells interact and interrogate MHC I complexes from different angles with different outcomes. In several examples, the outcome of these interactions is that target cells that are sensitive to killing by NK cells are resistant to killing by T cells, and the opposite is true, leading to the seminal observation of the “missing self-hypothesis” by Karre.^47,96^ T-cells, via their TCRs, recognize foreign peptides presented by MHC I complexes and get an activation signal (signal-1) from antigen-presenting cells (APCs) and cancer cells or virus-infected cells. T-cells ignore MHC I-presenting self-peptide or cells with low MHC I expression, such as some virus-infected cells, and delegate this function to NK cells. Additionally, T-cells do not tolerate polymorphism in the HLA groups that compose MHC I complexes and, as a consequence, mediate tissue rejection and destruction. In contrast, NK cell interaction with MHC I induces a tolerogenic signal via inhibitory signals from interacting killer cell immunoglobulin-like receptors (KIRs), and NKG2A/CD94.^97,98^ Additionally, NK cells tolerate allogeneic variability and polymorphism in HLA^99,100^ to a certain degree. However, they always interpret MHC I absence in scrutinized cells as non-self that must be destroyed.^47,101^ Indeed, NK cells were shown to kill preferentially cells lacking MHC I.^102,103^ NK cells will also destroy cells presenting certain empty MHC I complexes lacking a self-peptide.^104^ Others reported protection from lysis by empty MHC I.^105^ However, empty MHC I is unlikely to be expressed at the cell surface as loading the peptide onto MHC I complex is a requirement for quality control before export to the cell membrane,^106^ and this expression is only seen at temperatures near 26 °C in the absence of TAP (transporter associated with antigen processing).^107^ NK cells may also kill cells due to mutations in the peptides presented by certain HLA molecules, which may affect the interaction between KIRs and target cells, influencing NK cell activity.^108–110^

The inhibitory arm involves primarily KIRs with long cytoplasmic domains KIR-2DL, KIR-3DL, or C-type lectin receptors CD94/NKG2A/B interacting with MHC I complex. Inhibitory receptors CD94/NKG2A/B in normal cells recognize HLA-E molecules presenting the leader sequence peptides of the HLA-A, HLA-B, and HLA-C groups. Furthermore, HLA-E becomes only expressed at the cell surface when occupied by these peptides. This recognition of normalcy in cells inhibits NK cells only when they express normal levels of classical HLA class I molecules, effectively preventing NK cell-mediated cytotoxicity against normal cells.

**Fig. 2:**
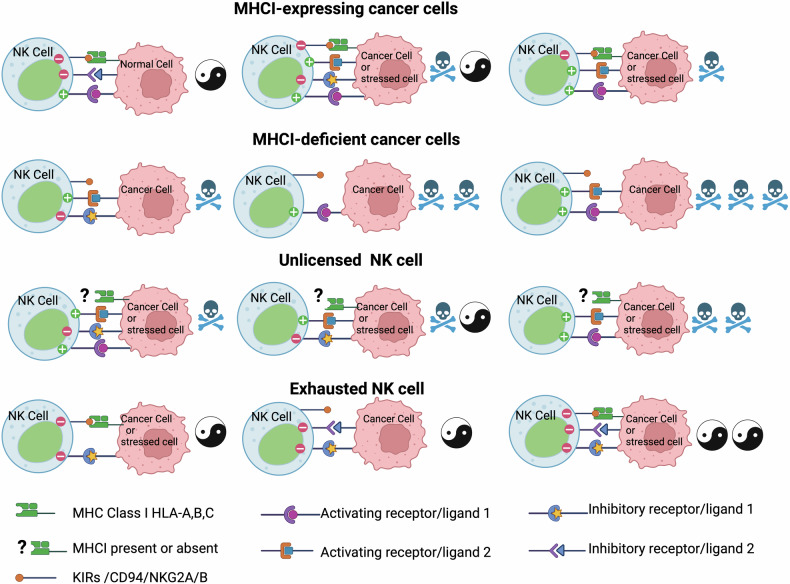
MHC I and the balance of stimulations and inhibitions dictate rules of engagement with cancer and stressed cells. Cancer and stressed cells expressing MHC I usually have multiple triggering ligands and can only escape NK cells if the balance of inhibitory signals is higher than activation. Cancer cells deficient in MHC I are killed through the “missing self” rule and are unlikely to escape NK cells, especially if the signaling balance favors activation. Unlicensed NK cells cannot kill through the “missing self” rule because they lack KIRS /CD94/NKG2A/B but are most likely to kill cancer and stressed cells that induce reasonable stimulation of NK cells due to the missing MHC I inhibition. Exhausted NK cells, usually having a dominance of inhibitory receptors, are less likely to kill cancer and stressed cells

Therefore, MHC I recognition is the primary and default inhibitory mechanism through which NK cells decide to engage scrutinized cells. Thus, the lack of MHC I recognition by KIRs, which exposes the missing self,^96^ is one of the main and default regulators of NK cell killing (Fig. 2). NK cells achieve optimal functionality through KIRS interactions with the four MHC I classes during their development when NK cells are educated or licensed.^111^ Tumorigenesis is characterized by reduced MHC I expression.^112,113^ MHC I deficient cancer cells can escape T-cells, but not NK cells, as these are MHC I unrestricted cells. However, MHC I deficient cancer cells may still escape NK cell surveillance due to other dysfunctions. This escape is mainly mediated through the anergy of NK cells due to weak activation or exhaustion, which can be reversed by cytokines such as IL-18 and IL-12.^114^

Overall, the interactions of NK cells and T-cells with MHC I are quite similar but yield different outcomes. NK cell interrogation of MHC I creates a tolerance signal that accepts HLA polymorphisms unless HLA is completely missing, very polymorphic, or from another species. This tolerance signal is relevant to fetus implantation, transplantation, and rejection and is evident in the urochordate *Botryllus. Schlosseri*, the closest invertebrate to vertebrates, which has only NK cells with no T or B-cells.^115–117^ Each *B. Schlosseri* individual transplants daily with others to form chimeras, and each need only one common allele of Botryllus histocompatibility factor^118–120^ to transplant with another individual successfully. The *B. Schlosseri* histocompatibility complex allowing this transplantation has extensive polymorphism^119^ and the mechanism that controls the tolerance signal and success of transplantation is mediated by BsCD94-1gene, a CD94-related transmembrane receptor of vertebrate NK cells, expressed on the surface of a subpopulation of Botryllus blood cells and upregulated during the allorecognition process.^121^ CD94 is expressed in modern NK and CD8 T-cells to interact with non-classical MHC I HLA-E, presenting the leader sequence peptides of HLA-A, HLA-B, and HLA-C groups. CD94 associates as a heterodimer with NKG2C and DAP12 to activate NK and T-cells or with NKG2A to inhibit them. This suggests first that NK cells are more ancient than T-cells and second that original NK cells via MHC I may have been designed initially to identify the self but also to regulate asexual reproduction and tolerance between two close individuals.

## NK strategies to identify the self

NK cells utilize two strategies to identify the self through MHC I. In one strategy, they recognize polymorphic MHC I proteins using polymorphic KIRs. In another strategy, they utilize the CD94-NKG2 receptor to query HLA-E, presenting conserved peptides derived from all HLA-A, B, and C classes. Both signals synergize to further prevent NK cells from killing normal cells. KIRs interactions with the four MHC I classes have been solved by crystallography.^122–124^ Structural analysis shows the two immunoglobulin-like extracellular domains of KIRs, D1 and D2 (in KIR2D receptors), to be arranged, depending on KIR members, like two hands (V-shaped) with angles between 66° and 81° and with each hand slightly twisting (along the axis of D1 or D2) at the wrist (hinge). This opening of the angle was found to affect the affinity of KIRs to HLA-C ligands.^125^ Near the KIR’s wrist is placed the presented peptide in a groove between the α1 and α2 helices of HLA. At this KIR-peptide-HLA interface, on the KIR side, D2 interacts with a well-conserved docking region of the HLA α2 helix spanning from amino acids 145 to 151. On the other hand, the regions of interaction between D1 of KIR and α1 helix are variable and seem to determine the specificity for each KIR. KIRs exhibit a high degree of polymorphism in humans, with a number of 2238 alleles reported in 2024 (https://www.ebi.ac.uk/ipd/kir/). This genetic diversity is “the single most important factor that shapes functional NK cell repertoires”.^126^ As an example of KIRs diversity in a defined population, a recent study reported using 1173 individuals of Japanese descent, 118 KIR alleles in 13 genes.^127^ The high diversity of the 16 different KIR genes on chromosome 19q13.4 is promoted by their head-to-tail orientation, which facilitates the deletion or duplication of *KIR* genes. KIRs are categorized into two haplotypes: A, which mainly encodes inhibitory KIRs, and B, which encodes stimulatory KIRs. The number of KIR genes per individual varies on different haplotypes and ranges from six to sixteen genes. As a rule, a particular KIR gene in an individual will be expressed stochastically in some NK cells, leading to subsets of NK cells within a person expressing different combinations of KIR receptors, with a majority not exceeding two. This stochastic expression increases the diversity of NK cells, with some NK cell subsets having only inhibitory and other subsets only stimulatory KIRs.^128^ KIR2DL4 is present in all haplotypes and is exceptionally expressed in all individuals. HLA-G, a non-classical HLA class I molecule, specifically expressed in extravillous trophoblasts is the only known ligand of KIR2DL4, and as we will see later, plays a major regulatory role in maternal-fetal immune tolerance and is also highly expressed in tumors.

An important difference between activating and inhibitory KIRs is that despite the high homology of their extracellular domains their binding to MHC I is weaker compared to inhibitory KIRs. KIRs that transmit inhibitory signaling have longer intracellular domains containing an immunoreceptor tyrosine-based inhibitory motifs (ITIMs), which associate with phosphatases like SHP-1. In contrast, KIRs that transmit activating signaling have a short intracellular domain containing an immunoreceptor tyrosine-based activating motif (ITAM) that associates with activating adapter DAP12 to signal through Syk/ZAP-70 tyrosine kinases. An exception to this rule is KIR2DL4, which is a long-tailed but activating KIR that associates with FcεRI-γ instead of DAP12.^129^ Although KIR2DL4 is defined as an activating KIR, its association with ligand HLA-G does not lead to more NK cells cytotoxicity but rather to cytokine secretion^130^ as do dNK cells. KIR2DL4 expression at the cell surface is restricted to cytokine-producing CD56^bright^ and is not detected on CD56^dim^ NK cells surface but interestingly did so after cell culture in vitro.^131^ However, KIR2DL4 is also located intracellularly in the endosomes of CD56^dim^ primary NK cells, where it can be activated by soluble HLA-G.^132^ This endosomal signaling by KIR2DL4 activates NF-κB and AKT, leading to IFNγ secretion.^133^ HLA-G can also be transferred to NK cells via endocytosis^134^ and trogocytosis, leading to a state of tolerance without compromising the antiviral response.^135^ This induced state of tolerance could also drive tumor resistance to therapies and affect the tumor microenvironment.^136^ KIR2DL4 fulfills its inhibitory receptor role when bound by HLA-G (soluble, membrane-bound bound, or trogocytosed). This triggers the phosphorylation of KIR2DL4, ITIM domain, leading to the recruitment of SHP-2 and the dephosphorylation of downstream signaling activating molecules and decreasing NK cell cytotoxicity. However, due to a positively charged Arginine on its transmembrane domain, KIR2DL4 can associate with FcεRI-γ.^129^ This association leads to the phosphorylation of the ITAM on FcεRI-γ, thus allowing NK cells to produce cytokines, including IFNγ, even though the cytotoxic response is generally suppressed due to the ITIM in KIR2DL4. The role of inhibitory KIRs is to interpret a “do not kill me” signal from HLA presenting a self-peptide, while activating KIRs are to interpret a “kill me” signal from HLA presenting specific viral peptides^137^ or open HLA with no peptides.^138,139^ The protective role of activating KIRs against certain viral infections has been reported for KIR3DS1^+^ NK cells against HIV-1^140^ and H1N1 influenza.^141^ However, activating KIRs could also prolong inflammation and injury, as in chronic hepatitis,^142^ and as we will see later, KIR composition could also affect autoimmunity.

## Maturation and education of NK cells (the pre-2024 view)

**Fig. 3:**
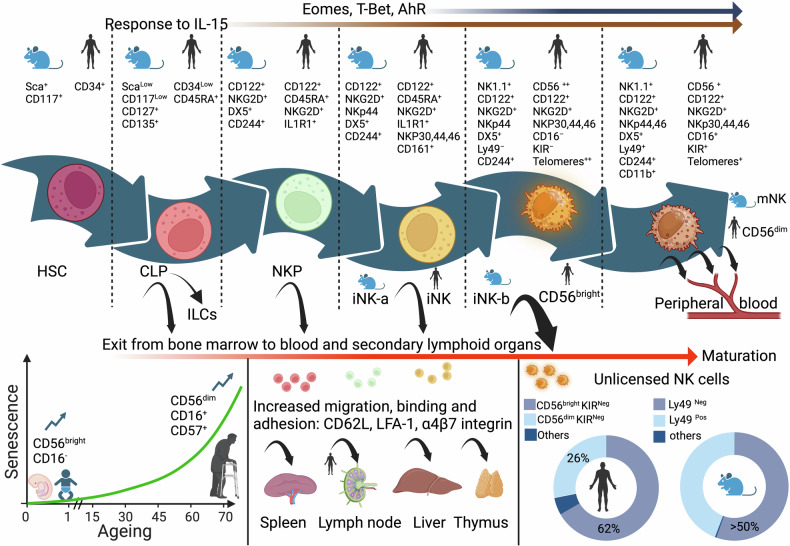
Development and maturation of NK cells. A pre-2024 view. Starting from a CD34^+^ hematopoietic stem cell in the bone marrow to a fully functional and mature CD56^pos^ CD16^pos^ NK cell. NK cell development and maturation (left to right axis) is marked by the acquisition of cytokine receptors responding to IL-15 transcription factors EOMES, T-bet and AhR and the acquisition of inhibitory receptors KIRs and antibody-mediated cytotoxicity receptor CD16. Due to increased adhesion proteins, NK cells could be released earlier than expected and migrate to secondary lymphoid organs to continue their varied maturations and education. NK cells released earlier CD56^bright^ KIR^Neg^ and CD56^dim^ KIR^Neg^ are unlicensed and their proportions in humans and mice are substantial, suggesting an evolutionary advantage to unlicensed NK cell release from the bone marrow, which is frequent at younger age and subsides to favor fully mature NK cells in older adults

The earliest NK progenitor was described in the bone marrow of mice^143^; consequently, bone marrow ablation results in NK cell deficiency. NK cells mature and receive an “education” or “license” early in the bone marrow (Fig. 3). This process is designed to increase their reactivity threshold by experiencing inhibitory signals from self-MHC I. Indeed, the capacity of a future mature NK cell to respond to stimulation is quantitatively determined by the strength of inhibitory signals received from MHC I molecules during NK cell education.^144^ Uneducated NK cells respond to inhibitory signals with strong production of phosphatase SHP-1, leading to their rapid inactivation, while educated, licensed NK cells have reduced SHP-1 production when encountering these inhibitory ligands, allowing them to remain activated.^145^ Therefore, educated NK cells are more cytolytic, and their maturation starts from a CD34^+^ human hematopoietic stem cell or mouse Sca^+^, CD117^+^ to the common lymphoid progenitor, which expresses IL2Rβ, responds to IL-15,^146^ and maintains this expression throughout the maturation stages, branching into an intermediary natural killer precursor (NKP) committed to developing into NK lineage which develops first into an immature iNK cell and then a mature NK cell with a CD56^bright^ phenotype that upon further maturation becomes CD56^dim^ ^147^ in humans. In the mouse, the NKP precursor develops into an immature iNK-a then an iNK-b stage, which is closer to human CD56^bright^ stage with further maturation by acquisition of Ly49. In humans, this phylogeny is supported by the longer telomeres found in CD56^bright^ compared to CD56^dim^.^148,149^ Although they are functionally similar in their interaction with MHC I. There are significant differences between mouse and human NK cells at the level of markers, residency, and longevity. For example, human NK cells can be expanded in vitro for extended periods of time, while mouse NK cells always die after a few weeks in culture. Similarly, opposite to humans, mouse NK cells are seldom found in the lymph nodes and mouse NK do not express CD56. Gradually, during their development, human NK cells acquire their receptors, starting with inhibitory CD161, then adhesion molecule CD56, inhibitory CD94/NKG2A, and activation receptors NKp46 and NKG2D. Acquisition of inhibitory and activating KIRs and later, CD16 complete their maturation.^150–152^ Transcription factor EOMES plays a role in early NK cell maturation and enhances CD16 expression, while T-BET controls maturation markers and induction of KIR expression.^153^ However, it is essential to note that maturation and education could be carried out in lymph nodes, thymus, uterus, liver, and mucosal lymphoid tissues, probably for cells that drop early of the bone marrow education before the maturation of CD56^bright^ to CD56^dim^ and the acquisition of CD16 and KIRs. Interestingly, the proportion of CD56^bright^ CD16^neg^ is higher in fetal tissues,^154^ and this population also decreases with age while CD56^dim^ CD16^pos^ increases.^155^ This suggests that maturation and education of “dropout” NK cells at the CD56^bright^ stage and earlier is high at a young age in the bone marrow and is reduced in the elderly. This might be due to the age-related decline of the secondary lymphoid sites, such as the thymus^156^ and lymph nodes.^157,158^ Possibly, at a younger age, these secondary sites might be more able to recruit less mature NK cells and induce them to exit the bone marrow early.

Like the stromal cells of the bone marrow, which provide necessary cytokines such as IL-15 and IL-7 for NK maturation,^159^ the stromal cells found in secondary lymphoid sites such as the spleen can also provide these cytokines.^160^ However, secondary lymphoid sites contain other monocyte populations like DCs, which might provide additional cytokines such as IL-2 and IL-15.^161^ Since mouse models have shown that bone marrow ablation results in NK cell deficiency, it can be assumed that any NK cell maturing in a secondary lymphoid organ is originally from the bone marrow regardless of its maturation stage. Indeed, upon exiting the bone marrow at the earliest NKP stage, these cells can be found transiting in PB among the CD34^+^ hematopoietic stem cell population. Not surprisingly, CD34^+^ NKP cells in lymph node highly express surface proteins, CD62L, lymphocyte function-associated antigen 1 (LFA-1), and α_4_β_7_ integrins, allowing cell migration, high binding, and rolling adhesion.^162^ It is unclear if NK cells that exit the bone marrow at early stages can be licensed elsewhere or if they remain unlicensed without acquiring KIRs. Both humans and mice present a large population of unlicensed NK cells without KIRs or Ly49, respectively. In humans, 62% of CD56 ^bright^ NK cells lack KIRs, while 26% of CD56 ^dim^ NK cells don’t express them, suggesting a large population of circulating NK cells is unlicensed^163^ and that more CD56^bright^ exit the bone marrow earlier. Similarly, 50% of NK cells in mice are Ly49 negative and unlicensed.^111^

Interleukins IL-12, IL-15, and IL-18 play a significant role in NK cell maturation and can reeducate unlicensed NK cells to enhance their functionality and exert stronger responses.^164^ KIRs acquisition by unlicensed human KIR^Neg^ that are CD56^bright^ and CD56 ^dim^ NK cells can be obtained after stimulation with IL-15 in the presence of stromal cells.^165^ Similarly, de novo expression of KIRs and NKG2A in unlicensed NK cells can be obtained using IL-2, IL-15, or IL-12 only.^164,166^ These observations have an important impact on immunotherapies using primary NK cells. Moreover, NK cells infiltrating solid cancers have been reported to be predominately CD56^bright^.^167^ Therefore, it is essential to understand how these unlicensed NK populations operate compared to licensed ones and if licensing is required for NK cells to carry out their functions.

In a tumor environment characterized by reduced MHC I expression,^112,113^ the fate of cancer cells facing licensed NK cells is almost certainly death and will be influenced by the balance between activators and inhibitors on their surface (Fig. 2). If NK cell activation by MHC I deficient cancer cells is weak or the balance of inhibitory signals is high, leading to anergy and exhaustion of NK cells, then activation by cytokines such as IL-18 and IL-12 may restore their activation.^114^ However, licensed NK cells in an MHC I sufficient environment will be inhibited, especially without activation or with increased inhibition from cancer cells. This exact experiment was reported using MHC I deficient cell line RMA-S and MHC I sufficient RMA cell lines grown subcutaneously in the same mouse. It showed better control of MHC I deficient RMA-S tumors.^47^ This suggests that the MHC I expression could offer an escape mechanism from licensed NK cells in the absence of a convincing activation that could override MHC I inhibition. However, this escape is unlikely with unlicensed NK cells that don’t express KIRs. Indeed, KIR-deficient unlicensed NK cells are more efficient than licensed NK cells at killing MHC I sufficient RMA cells.^168^ Similarly, the blockade of KIRs enhanced ex-vivo patient-derived NK cell cytotoxicity against multiple myeloma.^169^ Therefore, unlicensed NK cells offer an evolutionary advantage against the narrow NK specialization and broaden the spectrum of action for NK cells instead of relying on one rule regarding MHC I status. This is even more obvious in the case of viral infection against which NK cells are essential, where particularly unlicensed NK cells offer an edge. Viruses can alter MHC I antigen presentation in an attempt to escape T-cells.^170^ MHC I alteration leading to its downregulation does not escape licensed NK cells. However, few viruses, such as MCMV, express mimics of MHC I that bind to Ly49, the equivalent of KIRs in mice, and mediate repression of NK cell function.^171^ Immunological synapses initiated by NK cells when in contact with cancer cells are inhibited by KIRs.^172^ Since unlicensed NK cells do not express inhibitory KIRs but express activating KIRs, the binding by the viral MHC I mimics to activating KIRs leads to the activation of NK cells, making them instrumental in resisting MCMV infection. There is an evolutionary advantage to having polyfunctional populations of licensed and unlicensed NK cells that can be CD56^dim^ or CD56^bright^ with numerous phenotypes estimated in the thousands, maturing and receiving different “educations” in the bone marrow or second lymphoid organs. This advantage is apparent when facing threats that use evolution as a mechanism to adapt.

### Maturation and education and the new view on the origin of NK lineages

**Fig. 4:**
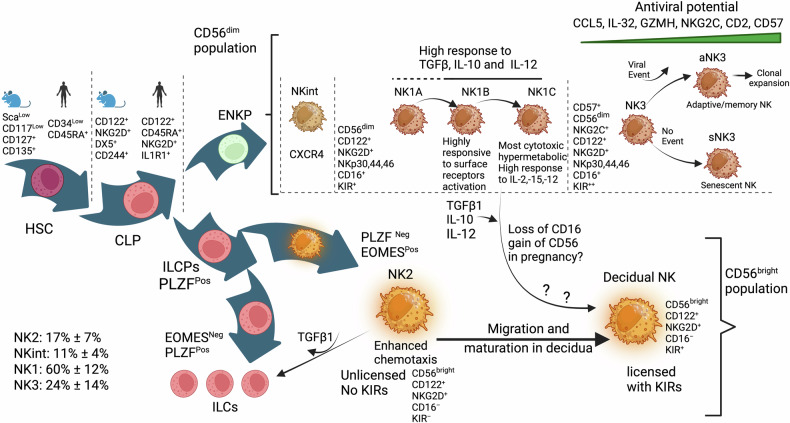
Development and maturation of NK cells. A 2024 view. NK cells originate from two lineages. An early natural killer progenitor (ENKP), which produces the CD56^dim^ population, and another progenitor deriving from an innate lymphoid progenitor (ILCP), which produces both CD56^bright^ and also ILCs. Both ENKP and ILCP would originate from a common lymphoid progenitor (CLP). The ENKP derived CD56^dim^ population matures, after an intermediary stage NKint, into an NK1 subset composed of three subsets: NK1A, NK1B, and NK1C with increased maturation but differing phenotypes related to response to surface receptors (NK1B), cytokine response and increased cytolytic activity (NK1C). A later more mature stage NK3 is characterized by increased CD57 expression, suggesting an adaptive phenotype with high NKG2C and antiviral potential that may lead to clonal expansion of adaptive/memory cells or may lead to senescence if no viral event occurs. The CD56^bright^ less mature population (NK2) is characterized by enhanced chemotaxis and is unlicensed with no KIRs and no CD16. NK2 subset is probably the source of dNK cells in pregnancy after migrating to the uterus. NK1B subset’s high response to TGFβ, IL-10, and IL-12 suggests it may contribute to building dNK populations with the potential to reduce the NK1C subset

The Common lymphoid progenitor (CLP) can generate, in addition to committed NK cells, Innate lymphoid cells^173^ (ILCs) (Fig. 4). These are mostly tissue-resident innate immune cells without cytolytic activity and are subdivided into three groups. The ILC1s group when stimulated by IL-12, IL-15, and proinflammatory IL-1b will produce IFNγ, without cytolytic function, termed type 1 immunity, and participate in viral and bacterial infection. ILC2 group function is type 2 immunity and responds to parasites such as helminths and allergens when stimulated by IL-25, IL-33, and TSLP. The ILC3 group mediates type 3 immunity in response to microbes, such as bacteria, by producing, among others, antimicrobial peptides when stimulated by IL-1b and IL-23. In mice both ILC1s and NK cells produce IFNγ, are both NK1.1^+^, NKp46^+^, CD3ε^−^ and express transcription factor T-bet.^174^ Commitment to an ILC progenitor (ILCP) lineage but not NK lineage requires the expression of transcription factor PLZF.^175^ However, ILCP co-expressing PLZF and Inhibitor of DNA binding 2 (ID2) retain the potential to produce an NK cell lineage suggesting a common ancestor of ILC1s and NK cells.^176^ Both ILC1s and NK cells express T-Bet. However, ILC1s do not express EOMES, while it is essential for NK cell development in the bone marrow.^177,178^ Using single-cell sequencing, two very recent studies aimed to understand how NK cells that appear after birth, originate and differentiate from ILC1s group, which are present in fetal life and beyond. In one study, Liang et al. show the expression of both PLZF and EOMES to confer both an NK and ILC1s potential and that NK-committed precursor cells express Eomes^high^ ^93^ but not PLZF and that the expression of Eomes transcription factor precludes the development of ILC2 and ILC3 groups. In the other study, Ding et al.^94^ identified two NK-committed lineages. One from an early NK progenitor (ENKP), developing into Ly49H^+^ NK cells and an ILCP-derived NK lineage with low expression of Ly49H. Both studies identify NK-committed lineages in the bone marrow, which may represent different stages of NK progenitor development. Eomes expression is, therefore, intrinsic to the NK phenotype, and the higher Eomes expression is, the closer to the mature phenotype NK cells are.

In human PB, Vivier et al.^41^ delineated three major subsets of NK cells discernible through single-cell transcriptomic analysis. One subset, called NK2, is CD56^bright^ and CD16^neg^, along with ID2 expression, and lacks KIRs, suggesting an immature phenotype. This subset showed markers of tissue residency. The most abundant subset in the blood, called NK1, is CD56^dim^ CD16^pos^, which expresses KIRs, GZMA, GZMB, and PRF1, a phenotype that suggests maturity. A third subset, termed NK3, is NKG2C^high^, CD16^dim^, CD57^pos^, suggesting further maturation and an adaptive phenotype. Of note, adaptive NKG2C^high^CD57^+^ cells expand in humans infected with HCMV.^179,180^ The study concludes that the two populations, NK1 and NK3, are originating from ENKPs, and that NK2 cells originate from ILCPs.

It is unclear whether these three populations exhibit plasticity and can convert into one another. However, there are reports of conversion from a CD56^dim^ to CD56^bright^ phenotype under IL-12.^181^ Interestingly, TGFβ can convert PB CD16^pos^ into a CD16^neg^ decidual like NK cells,^182^ and NK cells exposed to TGFβ or its relative Activin, acquire a gene signature and phenotype similar to the less cytotoxic ILCs, becoming unable to control tumor growth in mice.^183–185^ This suggests IL-12 and TGFβ1 may be possible mechanisms for converting NK1 to an NK2-like state or NK2 to an ILC state. Of the three subsets composing NK1 (NK1A, NK1B, and NK1C), NK1B appears the most likely to convert to an NK2-like or decidual phenotype as it has a strong response signature to IL-12, TGFβ, and IL-10.

The NK1 subset with further maturation leads to the NK3 phenotype, with increased KIRs and high CD57 expression. CD57 is associated with more experienced and terminally differentiated NK cells, possibly on the verge of senescence^186^ with higher frequency in older age.^187^ CD57 is also a marker of senescent T-cells that have short telomeres and low replication potential.^188,189^ NK3 population might specialize in highly effective and adaptive properties with memory-like features if they encounter an event such as viral infection. In the absence of such an event, they could become terminally senescent. This antiviral phenotype is suggested by the gradual increase from NK1 to NK3 of Granzyme H, which destroys critical adenoviral viral proteins that inhibit granzyme B, which is also present in NK3.^190^ Granzyme H also destroys the La-mediated HCV-IRES translational activity.^191^ Similarly, the exclusive expression of CCL5 in NK3 suggests antiviral defenses against Influenza A virus.^192^ Moreover, IL-32, which is elevated in NK3, plays a crucial role in responding to infections caused by viruses like HIV-1 and influenza. Additionally, it provides protection against cell death induced by the vesicular stomatitis virus. Notably, IL-32 exhibits antagonistic effects against the DNA virus HSV-2 in both epithelial Vero cells and human umbilical cord endothelial cells, thereby influencing the production of HSV-2,^193^ Finally, NK3 increased NKG2C expression reinforces the antiviral defense^194^ and NKG2C as well as ADCC mediated responses are enhanced by co-stimulatory molecule CD2^195^ which is also induced in NK3 subset.

Vivier et al. examined whether any subset is preferentially found in patients’ tumors and found the proportion of NK2 cells was increased in most tumors tested. NK2 population was characterized by higher CXCR3 expression, in agreement with better homing into tumors of CXCR3^+^ NK cells in a CXCL10-dependent fashion, leading to improved survival.^196^ However, CXCR2 and CXCR4 (distinguishing NKint and NK1A, respectively) were reported to enhance the migration of human primary NK cells to tumors expressing their ligands.^197^ NK1B cells high potential to respond to activation through increased surface receptors, suggest their potential in immunotherapeutic strategies. However, the NK1C subset’s pronounced cytoskeletal activity and cell-killing signature suggest it is the most cytotoxic. Overall, these studies suggest that an NK phenotype that is optimal for cancer immunotherapies may be within reach but still awaits further confirmation. Therefore, the lineage ENKP to NK1 might be the phase with the highest antitumor activity, while the further mature state NK3 excels in antiviral defenses. The lineage ILCP to NK2 appears to be mainly tasked with cytokine production and immunoregulatory functions like dNK. We can also infer that NK2 subset which is CD56^bright^ CD16^neg^ and KIR^neg^ is probably the seed of dNK cells that migrate to decidua in pregnancy, to mature and gain KIRs without gaining CD16.

## NK cell activation mechanisms that trigger killing

**Fig. 5:**
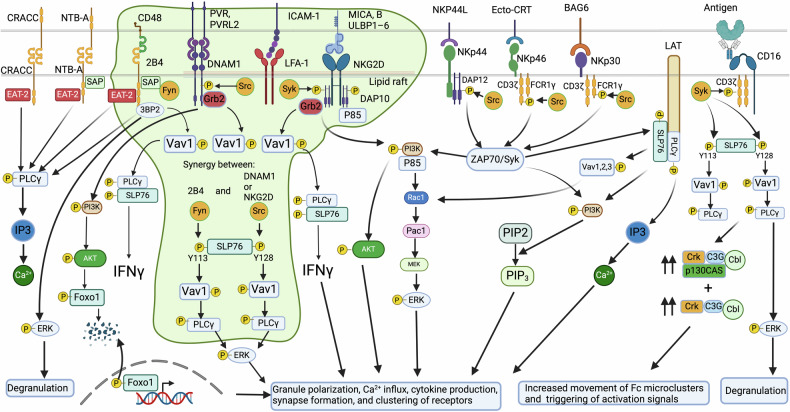
Dynamics of activation signaling in NK cells in contact with cancer and stressed cells. Activation signaling from Slam family 2B4, NTB-A, and CRACC. Upon ITSM phosphorylation, following ligand binding, an activation signal can be generated depending on the recruitment of EAT-2 and SLAM-associated protein (SAP), thereby blocking the binding site of lipid phosphatases SHP-1 and SHP-2. SAP recruits the Src-family kinase Fyn, leading to downstream PLCγ1, PLCγ2, and PI3K signaling. 2B4 can also recruit after phosphorylation, another adapter protein 3BP2, which activates VAV1 and ERK pathway upon phosphorylation. DNAM-1 engaged with ligand PVR or nectin-2 is tyrosine phosphorylated by Src kinases. This phosphorylation enables the binding of adapter Grb2 to DNAM-1, leading to VAV1, PI3K, SLP-76, and PLCγ1 activation, thereby increasing calcium fluxes and activating ERK and AKT pathways leading to FOXO1 degradation. DNAM-1 activating signal has a synergetic effect with LFA-1, to which it can be associated physically to induce tyrosine kinase Fyn to phosphorylate DNAM-1. NKG2D associates with adapter DAP10 after binding to ligand UL16-binding proteins (ULBP)1–6 and to ligands MICA and MICB, whose expression is regulated by the heat shock stress pathway or by DNA damage induced by chemotherapy and radiotherapy. NKG2D activation is triggered upon ligand engagement, leading to assembly with adapter DAP10 and phosphorylation of its motif followed by recruitment of PI3K, growth factor receptor-bound protein 2 (Grb2), VAV1, SLP-76, GTPase Rac1-dependent actin cytoskeleton rearrangement, thereby leading to MAPK signaling pathway activation and Pak1–Mek–Erk cascade signaling pathway. This culminates in granule polarization, calcium influx, cytokine production, synapse formation, and clustering of receptors. A parallel activation pathway triggered by PI3K is the AKT/mTOR pathway activation. NCR activation and killing depend on Src and Syk kinase activities. Engagement of NCRs with their cognate ligands induces associations with adapter CD3ζ, FCRγ or DAP12 whose ITAMs are phosphorylated by members of the Src kinase family: Lck, Fyn, Lyn, Fgr, Src, and Yes. The phosphorylated ITAMs will then attract and activate the tyrosine kinases Syk and ZAP70. These kinases will then phosphorylate other adapters, such as LAT, to recruit more downstream adapters and signaling complexes, such as PLCγ and PI3K, VAV1/2/3. Under PLCγ, Ca^2+^ influx increases, and PI3K will recruit p85, leading to phosphorylation of AKT and VAV1, which promotes GTPase Rac1-dependent actin cytoskeleton rearrangement, thereby activating the MAPK signaling pathway, leading to the Pac1–Mek–Erk cascade signaling pathway. The PI3K/AKT/mTOR pathway triggers a parallel activation pathway. CD16 is the only receptor in NK cells that can trigger alone and with the homodimer of adapters CD3ζ or FCRγ, an effective activation signal mediating antibody-dependent cellular cytotoxicity (ADCC). Only CD16 activation can lead to phosphorylation of both tyrosines (Y128) and tyrosine (Y113) on SLP-76. This double phosphorylation allows the binding of two VAV1 and more robust downstream signaling. Complexed Crk is required for CD16 signaling and the movement of microclusters of CD16 ligands on the lipid bilayer

**Fig. 6:**
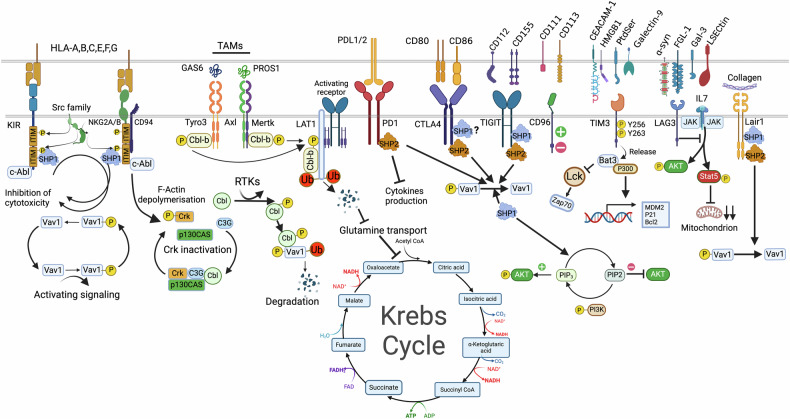
Dynamics of inhibitory signaling to block NK cell activation. Inhibitory receptors, including MHC class I-specific inhibitory receptors, target VAV1 for dephosphorylation by Src homology 2 domain-containing protein tyrosine phosphatase 1 SHP1. Another potent inhibitory relay is Crk dissociation mediated by c-Abl phosphorylation of Crk, which in its active form (non-phosphorylated) is associated with the complexes c-Cbl/Crk/C3G and p130CAS/Crk/C3G. C-Abl phosphorylation of Crk causes its dissociation from these complexes. Inhibitory signaling by CD94-NKG2A binding to HLA-E uses the E3 ubiquitin ligase c-Cbl to enhance the degradation of phosphorylated VAV1 and its downstream signaling PLCg2. Receptor tyrosine kinases TAM receptors (Tyro3, Axl, and Mertk) are expressed by multiple immune cells, including NK cells. TAM receptors phosphorylate ubiquitin ligase Cbl-b and dampen NK-cell activation signaling by promoting the degradation of LAT1, thus blocking VAV1-dependent signaling and, blocking, among others, glutamine transport and the fueling of the tricarboxylic cycle. DNAM-1 inhibition occurs when PD-1 recruits SHP2 to inhibit DNAM-1 phosphorylation via its intracellular domain signaling. TIGIT induces inhibitory signaling, while on the cancer cell side, PVR interaction with ligands TIGIT or DNAM-1 leads to tyrosine phosphorylation of the PVR’s ITIM domain by Src kinases and recruitment of SHP-2 followed by dephosphorylation of focal adhesion kinase and paxillin thereby reducing adhesion, increasing motility, survival, and proliferation of cancer cells. PD-1, CTLA-4, and TIGIT all recruit SHP-1 and SHP-2 leading to VAV1, PIP3 and SLP76 dephosphorylation. TIM-3 inhibition leads to Bat-3 release, which inhibits Lck and Zap70 activation and promotes with P300 the transcription of antiproliferative genes. LAG3 inhibition blocks STAT5 activation and reduces mitochondrial mass. Lair-1 inhibition by tumor collagen leads to SHP-1 and SHP-2 docking, VAV1 dephosphorylation and inactivation of NK cells

NK cells exhibit rapid activation and launch cytotoxic attacks on stressed, senescent, virus-infected, and cancer cells, bypassing the need for prior antigen presentation by MHC I. Unlike T and B-cells, which express specific activating receptors, NK cells express all activating and inhibitory receptors, creating an intricate and complicated equilibrium between multiple activating (Fig. 5) and inhibitory signals (Fig. 6) arising from their interaction with ligands on target cells with, however, a dominance of inhibitory receptors.^198^ It is important to note that except CD16, no other single activating receptors, including NKp46, NKG2D, 2B4, DNAM-1 (CD226), or CD2, are sufficient to activate NK cells on their own.^199,200^ Additionally, unlike most inhibitory receptors, many activating receptors, including KIRs, have no proper cytoplasmic signaling domain and rely on associations with adapter molecules that have ITAMs, allowing the creation and transmission of activating signals.

### Natural cytotoxicity receptors

Among the most potent activating receptors in NK cells, CD16 is the only receptor in NK cells that can trigger alone, in association with the homodimer of adapters CD3ζ or FCRγ, an effective activation signal mediating antibody-dependent cellular cytotoxicity (ADCC). A process where NK cells destroy target cells coated with antibodies.^201^ Other potent activating receptors for NK cells lacking an activating cytoplasmic tail include the natural cytotoxicity triggering receptors (NCRs) (NKp46, NKp30, and NKp44)^202^ (Fig. 5). However, some NKp44 isoforms contain a cytoplasmic ITIM-like motif.^203^ NCR ligands are not expressed in normal cells but are induced in pathological conditions.^204^ NKp30^66^ is critical for NK interactions with DCs and binds to ligand B7H6 expressed exclusively on tumor cells,^205^ but is also transiently expressed by activated T-cells.^95^ NKp46 receptor^206^ was recently found to recognize externalized calreticulin (ecto-CRT) expressed during ER stress, virus infection, and senescence.^207^ NKp46 prevents metastasis^208,209^ and mediates cytotoxicity against cells that are otherwise resistant to NK cells through the secretory pathway and TRAIL.^210^

Both NKp30 and NKp46 use activating adapters CD3ζ or FCRγ. NKp44^211^ interacts with ligand NKp44L,^212^ and uses homodimers of activating adapter DAP12.^203^ NKp44 exists in three isoforms (NKp44-1, 2, and 3), with the cytoplasmic domain of NKp44-1 containing an ITIM-like domain (EILYHTVA). The expression of ITIM-bearing NKp44-1 inhibitory isoform has been reported to be detrimental to the survival of acute myeloid leukemia patients.^213^ However, its expression during pregnancy in dNK cells^214^ allows decidua vascularization, maternal-fetal tolerance, and antiviral resistance. In this context, trophoblasts expression of NKp44L proliferating cell nuclear antigen (PCNA)^215^ and ligation to NKp44 through HLA or exosomes inhibits dNK cells through the ITIM-like domain, inhibits IFNγ secretion, and reduces their toxicity. Similarly, three forms were described for NKp30 (A-C) with different cytoplasmic sequences due to alternative splicing. Forms A and B induce IFNγ, TNFα, and IL-12B, while form C induces IL-10.^216^ Additionally, soluble B7H6 (sB7H6)^217^ and BAG-6 (sBAG-6)^218^ downregulate or inhibit NKp30 signaling. sBAG-6 is detectable in high levels in Chronic lymphocytic leukemia patients at advanced disease stages. Surprisingly, NK cells were activated when BAG-6 was presented on the surface of exosomes.^219^ This suggests an imbalance between soluble and exosomal BAG-6 could promote CCL evasion. Moreover, NKp30 and NKp44 engagement with cancer cells can induce NK cell death via the upregulation of Fas Ligand in certain tumors.^220^ Surprisingly, overexpression of NKp44 in NK-92 was shown to inhibit activation after binding of NKp44 to PCNA, which is widely overexpressed in tumor cells.^221,222^

NCR activation and the ensuing killing largely depend on Src and Syk kinase activities.^223,224^ The engagement of NCRs with their cognate ligands will induce associations with adapter CD3ζ, FCRγ or DAP12 whose ITAMs are phosphorylated by many redundant members of Src kinase family: Lck, Fyn, Lyn, Fgr, Src and Yes. The Phosphorylated ITAMs will then attract and activate the tyrosine kinases Syk and ZAP70 (Fig. 5). These kinases will then phosphorylate other adapters, such as LAT (linker for activation of T cells or P36). LAT is tyrosine phosphorylated upon stimulation of NK cells through FcγRIII receptors following contact with target cells to recruit more downstream adapters and signaling complexes, such as phospholipase C (PLCγ), phosphatidyl- inositol-3-OH kinase (PI3K), and guanine nucleotide exchange factor VAV1/2/3. Under PLCγ, Ca^2+^ influx increases, and PI3K will recruit p85, leading to phosphorylation of AKT, and VAV1, which promotes GTPase Rac1-dependent actin cytoskeleton rearrangement, thereby activating the MAPK signaling pathway, leading to the Pac1–Mek–Erk cascade signaling pathway. Since AKT is a major downstream target of PI3K,^225^ a parallel activation pathway is triggered by the PI3K/AKT/mTOR pathway. All these events culminate in granule polarization, calcium influx, cytokine production, synapse formation, and clustering of receptors. CD59 is another activating receptor physically associated with NKp46 and NKp30. Its activation leads to tyrosine phosphorylation of CD3ζ chains associated with these NCRs.^226^

### NKG2D receptor

Another pivotal receptor involved in NK cell tumor and senescence surveillance, a member of the NKG2 family of receptors, is NKG2D. In humans, due to the lack of an activation domain in its cytoplasmic tail, NKG2D associates with adapter DAP10^227^ after binding to ligand UL16-binding proteins (ULBP)1–6^228^ and to ligands MICA and MICB,^69^ whose expression is regulated by the heat shock stress pathway^229^ or by DNA damage induced by chemotherapy and radiotherapy.^230^ NKG2D ligands are absent in normal tissues but widely expressed in many cancers, including colorectal and ovarian cancers.^231,232^ Experimental evidence shows that the inducible expression of surface NKG2D ligands in tumors effectively controlled their initiation or growth^233^ and that mice deficient in NKG2D could not control tumors.^234^ However, just as it is common for other receptors such as NCRs, NKG2D ligands are also shed in soluble forms: sMICA and sULBP2, which have inhibitory properties.^235^ This inhibition is exerted even in the presence of membrane NKG2D ligands. Soluble NKG2D ligands shedding by tumors is metalloproteinases-dependent^236^ and could lead to high levels of NKG2D ligands in the sera and the tumor microenvironment to the point that NKG2D ligands inhibition with antibodies could enhance CTLA-4 and PD-1 immune checkpoint blockades.^237,238^ Soluble sMICA and sULBP2 levels in the serum of patients with oral squamous cell carcinoma, melanoma, and NSCL correlated with disease progression.^239–241^

NKG2D activation is triggered upon ligand engagement leading to assembly with adapter DAP10 and phosphorylation of its motif Tyr-ILe-Asn-Met at Tyrosine followed by recruitment of PI3K, growth factor receptor-bound protein 2 (Grb2), VAV1, SLP-76, GTPase Rac1-dependent actin cytoskeleton rearrangement, thereby leading like in the case of NCRs to MAPK signaling pathway activation and Pak1–Mek–Erk cascade signaling pathway. This culminates in granule polarization, calcium influx, cytokine production, synapse formation, and clustering of receptors. Similarly to NCR activation, a parallel activation pathway triggered by PI3K is the AKT/mTOR pathway activation.

### The SLAM family of receptors

Other critical receptors initiating NK cell responses upon binding to specific ligands on target cells are receptors of the signaling lymphocytic activation molecule family (SLAM) that possess one or more immunoreceptor tyrosine-based switch motif (ITSM) in their cytoplasmic tails. These are 2B4 (CD244), which is activated by ligand CD48,^242,243^ self-ligand NK-T-B-Antigen (NTB-A),^244^ and self-ligand CRACC.^245^ Upon ITSM phosphorylation, following ligand binding, either an activating or an inhibitory signal can be generated depending on the recruitment of EAT-2^244^ and SLAM-associated protein (SAP), thereby blocking the binding site of lipid phosphatases SHP-1^246^ and SHP-2,^247^ which generally inhibit NK effector functions and cytokine release. SAP is also able to recruit the Src-family kinase Fyn.^248^ CRACC, however, can associate only with EAT-2 but not SAP, leading to an effective downstream PLCγ1, PLCγ2, and PI3K signaling.^249^ 2B4 can also recruit after phosphorylation, another adapter protein 3BP2, which upon phosphorylation, activates VAV1 and the ERK pathway.^250^

### DNAM-1 receptor

DNAM-1(CD226)^63^ is a crucial co-stimulatory receptor for NK cells with a prominent role in anti-tumor and anti-viral surveillance. DNAM-1 cytoplasmic tail contains an ITT-like motif (YVNY), which upon DNAM-1 engagement with ligand PVR or nectin-2 is tyrosine phosphorylated by Src kinases. This phosphorylation enables the binding of adapter Grb2 to DNAM-1, leading to the activation of VAV-1, PI3K, SLP-76, and PLCγ1, thereby increasing calcium fluxes and activating ERK and AKT pathways.^251^ DNAM-1 activating signal has a synergetic effect with LFA-1, to which it can be associated physically, to induce tyrosine kinase Fyn to phosphorylate CD226.^252^ Association with LFA-1 is important for DNAM-1 clustering in the immune synapse,^253^ after LFA-1 interaction with PTA-1, which, in turn, associates with actin-binding protein 4.1G, to associates with membrane-associated guanylate kinase homolog protein leading to clustering and transport of DNAM-1 to lipid rafts.^254^ DNAM-1 does not have an exclusive ligand and must compete for PVR (CD155) and nectin-2 (CD112)^78^ against other inhibitory receptors, including TIGIT, TACTILE (CD96), and PVRIG (CD112R). The dynamics of this fierce competition will be discussed later in some detail. However, by virtue of DNAM-1 having a higher affinity to PVR than to nectin-2, it is safe to assume that NK cytotoxicity will largely depend on PVR expression level, and indeed PVR is widely expressed in human cancers.^255–257^ Other important activating receptors include NKp80, which binds to activation-induced C-type lectin (AICL), CD28 which binds to CD80 and CD86, CD2 which binds to CD48 (also a partner of 2B4) and CD58; the KIRs with short cytoplasmic domains, KIR-2DS and KIR-3DS, and C-type lectin receptors CD94/NKG2C, and NKG2E/H/2F.

## The synergy between activating signals

2B4 activation can synergize with NKG2D or DNAM-1 at the level of PLC-γ and ERK phosphorylation (Fig. 5). This synergy was shown to be required to overcome the inhibitory signaling by CD94-NKG2A binding to HLA-E that controls VAV1 phosphorylation and its downstream signaling, PLCγ2.^258^ It was later discovered that SLP-76 needed to be phosphorylated once by NKG2D or DNAM-1 in one tyrosine (Y128) and a separate phosphorylation by 2B4 at tyrosine (Y113). Only CD16 activation can lead to phosphorylation of both tyrosines on SLP-76. This double phosphorylation allows the binding of two VAV1 molecules^259^ with more robust downstream signaling. An interesting aspect of NKG2D and DNAM-1 signaling is that the activation of NKG2D can block DNAM-1 activation through the induction of TIGIT expression and the inhibition of DNAM-1 signaling.^260^ This phenomenon was explained by the reduction in Pyk2 and Erk1/2 phosphorylation upon DNAM-1 engagement. However, AKT and VAV1 activation remained unaffected.^260^ This observation is substantiated by another group that reported a lack of synergistic effects when co-expressing both DNAM-1 and NKG2D in NK-92.^261^ However, the fact that VAV1 and AKT activations were not affected or, more accurately, not increased suggests that the early event of DNAM-1 activation did not proceed. Another recently described mechanism of DNAM-1 inhibition occurs when PD-1, via its intracellular domain signaling, recruits SHP-2 to inhibit DNAM-1 phosphorylation^262^ (Fig. 6). Since TIGIT is induced by NKG2D activation^260^ and since PD-1 and TIGIT were found to be co-expressed in CD8 T-cells of NSLCC patients,^262^ it is possible that both TIGIT and PD-1 induced by NKG2D activation, conspire together to inhibit DNAM-1 signaling in NK cells. The inability of DNAM-1 to synergize with NKG2D signaling and NK cell cytotoxicity suggests an overlap or a rheostat mechanism accepting an “either-or” pathway, which could be designed to avoid exhaustion when two pathways could hyperactivate NK cells. Indeed, co-activator 2B4, which synergizes with NKG2D, can also synergize with DNAM-1, but not simultaneously.^258,259^ These findings have profound implications for cancer immunotherapy aiming to exploit NKG2D and DNAM-1 and suggest that it is better to combine each one of them with other modalities, such as immune checkpoints, especially in the case of loss of expression of one of them.^262–264^

Many synergetic activating signaling in NK cells, such as NKG2D, DNAM-1, 2B4, NTB-A, and CRACC, converge on the phosphorylation of VAV1. And inhibitory receptors, including MHC I-specific inhibitory receptors, target VAV1 for dephosphorylation by SHP-1.^265^ Another potent inhibitory relay is Crk dissociation mediated by c-Abl phosphorylation of Crk, which in its active form (non-phosphorylated) is associated with the complexes c-Cbl/Crk/C3G and p130CAS/Crk/C3G. c-Abl phosphorylation of Crk causes its dissociation from these complexes (Fig. 6). Complexed Crk is required for CD16 signaling and the movement of microclusters of CD16 ligands on the lipid bilayer.^266^ Additionally, the inhibitory signaling by CD94-NKG2A binding to HLA-E uses the E3 ubiquitin ligase c-Cbl to enhance the degradation of phosphorylated VAV1 and its downstream signaling PLCγ2.^258^ Therefore, Cbl-b inhibition affecting Vav1 can only be overcome by synergistic signaling of multiple activating receptors.^258^ Receptor tyrosine kinases TAM receptors (Tyro3, Axl, and Mertk) are expressed by multiple immune cells, including NK cells. TAM receptors phosphorylate ubiquitin ligase Cbl-b and dampen NK-cell activation signaling by promoting the degradation of (Large Amino-acid Transporter 1) LAT1, thus blocking VAV1-dependent signaling^267^ and blocking, among others, glutamine transport and the fueling of the tricarboxylic cycle. It is accepted that VAV1 might be the point of convergence for various activating and inhibitory pathways, offering a rational and strategic switch to turn off NK activation and prevent the downstream activation cascade.^268^ Therefore, preventing VAV1 deactivation could provide a potent means to activate NK cells, with, however, the potential risk of higher toxicity to normal tissues.

## The interplay of inhibitory and activating signals: The TIGIT/PVR/DNAM-1 axis

Most successful cancer immunotherapies are achieved using activating cytokines and activating receptors or their activation domains. This suggests that additional activation signals can be integrated into preexisting ones to strengthen them and reduce existing inhibitions. At the cell surface, activating and inhibitory receptors interact with their cognate ligands. Often, these ligands are unique to an activator or an inhibitory receptor. However, multiple instances exist where both the activating and inhibitory receptors compete for the same ligand, often to the benefit of the inhibitory receptor signaling. For example, the competition for HLA-E, the most ancient of the six functional HLA class I genes, by the inhibitory receptor CD94/NKG2A (Kd = 0.8 μM) and activating receptor CD94/NKG2C (Kd = 5.2 μM).^269^ Similarly, the competition for CD80 between immune checkpoint CTLA-4 (Kd = 0.46 μM) and CD28 (Kd = 4 μM)^270^ or for CD86 (CD86–CD28 ~ 20 μM and CD86–CTLA-4 ~ 2 μM). Another more complex and striking example is illustrated by immune checkpoint TIGIT and activating receptor DNAM-1, which compete for PVR (CD155) and nectin2 (CD112). In this race, DNAM-1 loses as TIGIT has a higher affinity for PVR (Kd = 1–3 nM) than DNAM-1 (Kd = 119 nM).^271^ TIGIT extends its inhibitory dominance by interacting with other inhibitory ligands, Nectin2, Nectin3,^272^ and Nectin4.^273^ In addition to TIGIT, CD112R(PVRIG) also competes with DNAM-1 for Nectin2,^274^ while CD96^275^ and KIR2DL5^276^ compete for PVR against DNAM-1.

DNAM-1 does not have an exclusive ligand for its activation, thus giving competing inhibitory receptors a clear advantage. This example illustrates the roadblocks for efficient NK cell activation at the level of competing extracellular domains for ligands. However, an additional layer of complexity is added by the fact that TIGIT will disrupt DNAM-1 homodimer assembly at the cell membrane, preventing its activation.^277^ This thug of war continues at the level of intracellular domains signaling with PVR/TGIT signaling blocking AKT phosphorylation, thus stabilizing transcription factor FOXO1, which inhibits NK and T-cell activation and enhances immunosuppressive functions of T-regulatory cells.^278^ The exact opposite is produced by PVR/DNAM-1 signaling, which phosphorylates AKT and destabilizes FOXO1 by phosphorylation, promoting its nuclear exclusion and degradation, thus enhancing NK and T cell activation.^279^

It is safe to assume that if these signals are present in the same cell, the inhibitory PVR/TIGIT axis will probably dominate the PVR/DNAM-1 axis. Another recently described mechanism of DNAM-1 inhibition occurs when PD-1, via its intracellular domain signaling, recruits SHP-2 to inhibit DNAM-1 phosphorylation.^262^ This finding is critical since PD-1 and TIGIT were found to be co-expressed in CD8 T-cells of NSLCC patients, suggesting the need for dual inhibition of PD-1 and TIGIT immune checkpoints.^262^ In addition, several tumors develop strategies to downregulate activators, including DNAM-1 expression in NK cells.^280–282^ Overall, inhibition and activation signals are regulated first through fierce competition for ligands with different intrinsic affinities at the cell surface. However, the axis PVR/TIGIT signaling between NK cells and cancer cells is bidirectional. On the NK cell side, TIGIT induces inhibitory signaling. In contrast, on the cancer cell side, PVR interaction with ligands TIGIT or DNAM leads to tyrosine phosphorylation of the PVR’s ITIM domain by Src kinases and recruitment of SHP-2 followed by dephosphorylation of focal adhesion kinase and paxillin thereby reducing adhesion, increasing motility, survival, and proliferation of cancer cells.^283–285^ Therefore, it is conceivable that if exhausted NK cells cannot kill cancer cells, they could make them stronger through stimulation of PVR or other immune checkpoints, especially with the ability of some NK cell subsets to support angiogenesis.^286^

## Kinetics of killing

The rapid killing of cancer and virus-infected cells suggests that all effectors are available in NK cells and ready for immediate delivery. This killing largely depends on Src and Syk kinase activities.^223,224^ However, whether NK cells can kill multiple cancer cells at once or over time will depend on the presence of activating signals and sustaining cytokines. In a six-h assay, NK-92MI cell line, which produces a membrane-bound IL-2, can kill ten cancer cells serially.^287^ The authors noted that the first kill was slower than subsequent ones and that if cells are denser, the following killings are executed more rapidly, suggesting possible simultaneous killings. Short distances between target cells might encourage disengagement with the killed cell and engagement with a new target. We reported in NK-92 expressing IL-2 tethered to its receptor IL2Rβ a replenishment of granzyme and perforin stores after 3 h of exposure to PC-3 cells, suggesting serial killing.^288^ Cytotoxic T-cells have been reported to polarize lytic granules toward different cells and interact with multiple targets simultaneously.^289,290^ Another study found that human primary NK can kill four cancer cells serially but cannot engage simultaneously with two or more cancer cells.^291^ This suggests that primary NK cells activated by IL-2 cannot multitask and must disengage from a killed cell to kill a second one. This might be due to a missing component that allows multiple polarizations. The same study also reported increased killing by ADCC using Rituximab. However, this may be due to the efficient synapse formation initiated by antibody Fc binding. Without a novel cell target nearby, NK cells can remain attached to the dead cancer cells, which could deepen its activation via prolonged contact of activating receptors with their ligand in a manner already observed in the case of T-cells.^290^

## Migration patterns

Release of activated NK cells from the bone marrow following inflammation or infection allows NK cells to migrate to affected tissues to kill abnormal cells and create inflammatory conditions in preparation for an adaptive immune response.^292^ The first step in the extravasation of NK cells into tissues requires tethering to endothelial cells, and this is accomplished by LFA-1, expressed on CD56^bright^ and CD56^dim^ subsets, and L-selectin (CD62L), which is only expressed in CD56^bright^ subset.^293^ Therefore, L-selectin is a significant determinant in CD56^bright^ delocalization from PB towards tissues. CD56^bright^ cells migration in tissues is decelerated by downregulation of L-selectin by IL-2, IL-15, or TGFβ1 and accelerated by increased L-selectin expression under IL-12, IL-10, or IFNα.^293^ Chemokine ligands play a role in this relay by exerting attraction functions by binding to G protein‐coupled chemokine receptors. They play a significant role in immune cell recruitment into tissues, including tumors, by attracting cells expressing their cognate chemokine receptor. Depending on their resting or activated states, NK cells express heterogeneously the four groups of chemokine receptors for ligands CXC, CC, CX3C, and C. NK cells express receptors CXCR1, CXCR2, and CX3CR1.^294–298^ In the bone marrow, specific chemokines, such as CCL3, which binds to receptors CCR1, CCR4, and CCR5, regulate NK cell localization and induce migration to PB. In contrast, CXCL12 induces the accumulation of NK cells expressing high CXCR4.^299^ Breast cancer cells and tumor-associated stromal cells express high levels of CXCL12 to stimulate their proliferation and invasiveness in autocrine and paracrine modes.^300^ Tumors also secrete chemokines ligands to attract pro-tumorigenic cells such as myeloid-derived suppressor cells (MDSCs),^301^ T-regulatory cells,^302^ Tumor-associated macrophages,^303^ and tumor-associated neutrophils.^304^ Monocyte chemoattractant CCL2 (MCP-1), which interacts with CCR2, plays a prominent role in tumor angiogenesis, tumor cell survival, and the recruitment of immunosuppressive cells that will challenge immune cells, including NK cells in the tumor microenvironment.^305^ These pro-tumorigenic cells will be recruited through the CCR2, CXCR1 and CXCR2 axes. These cells create a tumor microenvironment that suppresses immune cell invasion of the tumor cells’ chemokine ligand secretion, which will directly enhance the growth and survival of cancer cells in the tumor microenvironment and promote metastasis.^306^ However, chemokines play a dual role and could promote anti-tumorigenic effects by attracting NK and T-cells expressing chemokine receptors CXCR3 and CXCR4. For example, overexpression of CXCR4 in NK cells improved tumor eradication of U87-MG glioblastoma secreting CXCL12.^307^ Migration of human primary NK cells to CXCR1, CXCR2, and CXCR4 ligands was reported.^197^ However, CXCR4 is also overexpressed in more than 23 human cancers and contributes to tumor growth, angiogenesis, and metastasis. This overexpression would naturally capture CXCL12 at the surface of cancer cells, an effect that would distort the gradient that attracts typically immune cells to tumors.^308^

Studies showed that CXCR3^+^ NK cells infiltrate tumors in a CXCL10-dependent fashion, leading to improved survival,^196^ while NK cells from CXCR3^−/−^ mice show impaired tumor infiltration.^309^ Similarly, inhibiting pro-tumorigenic chemokine signaling enhances the potential of anti-tumorigenic chemokines, as exemplified by the knockdown of transcription factor Snail, reducing the expression of CXCR2 ligands (CXCL1 and CXCL2), and MDSCs attraction to the tumor via CXCR2, leading to increased T-cell and NK cell numbers in tumors.^310^

CD56^bright^ and CD56^dim^ primary NK cells express CXCR1, CXCR3, and CXCR4.^311^ However, it is clear that PB NK cells probably have different subsets with different chemokine phenotypes and migration abilities and that there are differences between individuals in these populations.^311^ For example, the CD56^bright^ CD16^+^ NK cells were the predominant population responding to IL-8 (CXCR1,2) and fractalkine (CX3CR1),^197^ while others reported CXCR1 and CXCR2 to be highly expressed by cytotoxic CD56^dim^ NK cells.^296,312^

In addition to chemokine receptors, NK cells express other chemotactic receptors, such as ChemR23^313^ and CCRL2,^314^ which, by attraction to chemerin, recruit NK cells to colocalize with DCs in inflammatory sites. ChemR23 is also expressed on macrophages, adipocytes, and endothelial cells,^315–317^ suggesting they all colocalize with NK cells.

Human NK cells activated by IL-2 express SIPR1,4 and 5, a G-coupled receptor proteins chemoattracted to bioactive lipid Sphingosine 1-phosphate (S1P).^318,319^ Receptor SIPR5 is expressed by NK and DCs, suggesting their colocalization.^320^ In inflamed tissues, S1P levels increase to promote the retention of immune cells.^321^ NK cells were also shown to directly recruit conventional type-1 dendritic cells (cDC1), which are critical for antitumor immunity through the secretion of CCL5 and XCL1.^322^ Senescent cells in aging tissues secrete senescence-associated secretory phenotype (SASP) proteins, which are inflammatory cytokines with chemokines GM-CSF, CCL2, 3, 4, and 5, CXCL1, 9, 10, and 11, which attract immune cells including NK cells, macrophages, neutrophils, and DCs. These immune cells will remove senescent cells but may also kill neighboring cancer cells in the same inflammatory environment. Chemokines binding to chemokine receptors is followed by internalization and degradation, which reduces homing. This could be alleviated by upregulating chemokine receptors.^323–325^

## NK cells role in autoimmunity

Two major subsets of NK cells can be distinguished. CD56^bright^ CD16^negative^, which secrete cytokines, and CD56^Dim^ CD16^positive^, which are highly cytotoxic. However, NK cells that secrete IL-10 and possess immunosuppressive functions could form a third group with immunoregulatory functions. Autoimmune diseases arise from autoreactive T-cells and autoantibody-producing B-cells (plasma cells) against self-antigens. Autoreactive T-cells that escape thymic deletion^326,327^ are present in most healthy humans, and 55–75% of the repertoire generated by random immunoglobulin G gene rearrangement during early B cell development in the bone marrow is autoreactive and removed by two checkpoints.^328^ In the case of T-cells, central to autoimmune diseases is the role played by DCs,^329^ which migrate to lymphoid organs to present pathogen-derived antigens to antigen-specific T-cells. NK cells, particularly CD56^bright^ NK cells, can, by production of GM-CSF and CD154, induce CD14^+^ monocyte differentiation into DCs in RA and psoriatic arthritis but not osteoarthritis OA patients.^330^ Therefore, RA NK cells provide a local milieu for monocytes to differentiate into DCs and sustain the disease. This could also be exacerbated by IFNγ secretion, which promotes Th1 polarization of CD4^+^ T. Similarly, the interaction of NK cells with DCs induces IFNγ, especially from the CD56^bright^ subset, which expresses surface molecules CD62L, CCR7, and CXCR3.^331^ This suggests this subset may colocalize with DCs in secondary organs and other inflamed tissues. However, several studies showed that although NK cells can increase in RA, they are less cytotoxic and have decreased IFNγ production.^332,333^ Since these NK cells also produce pro-inflammatory cytokine GM-CSF, it has been proposed that NK contribution to inflammation in RA might be due to the attraction of neutrophils, thereby upregulating pro-inflammatory CXCL2, CCL3, and LTb4, that sustain immune cell recruitment into inflamed joints.^334^ The involvement of NK cells in other autoimmune diseases remains contentious. For example, in multiple sclerosis, it is thought that NK cells fail to remove myelin-reactive T-cells and fail to suppress autologous CD4 + T cells compared to healthy controls.^335,336^ In Systemic Lupus Erythematosus (SLE), notable reductions in peripheral NK cell number and cytotoxicity were observed.^337,338^ However, the role of NK in developing SLE has been established through a bidirectional interaction between NK and peripheral DCs. NK cells augment IFNα production by activated DCs,^339^ in turn, IFNα increases NK cell production of IFNγ,^340^ thereby establishing highly inflammatory conditions. Incidentally, SLE patients have higher levels of IL-15, which is also conducive to increased inflammation.^341^ In Type 1 diabetes mellitus (T1DM), which is due to the destruction of pancreatic β cells by CD8 T-cells, a systematic reduction in the number and cytotoxicity of peripheral NK cells was observed.^342,343^ It is noteworthy that NK cells are also impaired in type 2 diabetes, suggesting their reduced activity in both diabetes types is mainly related to glucose levels and that the prevalence of infectious diseases and malignancy in type-2 diabetes patients may be associated with NK cell impairment.^344^ However, NK cells that infiltrate inflamed islet cells^345^ might have a sinister role in the development of T1DM by killing virus-infected pancreatic β cells,^346^ which reduce their HLA-1 expression to escape T-cells but become targets of NK cells. The subsequent β cell killing by NK cells could lead to the exposure of autoantigens recognized by CD8 T cells.

We have mentioned earlier that KIRs are categorized into two haplotypes: A, which mainly encodes inhibitory KIRs, and B, which encodes stimulatory KIRs. A study examining the role of the KIR haplotype on NK cells reported that KIR A1 haplotypes were positively associated with T1D in the subset of patients without the high T1D risk HLA genotype.^347^ In these patients, inhibitory KIR A2 haplotypes were over-transmitted, and the stimulatory KIR B haplotypes were under-transmitted, suggesting haplotypes A are predisposing and stimulatory haplotypes B confer protection. From our perspective, we interpret this result as due to the restricted ability of NK cells with inhibitory KIR A2 haplotype to kill or suppress overactive CD8 T-cells thus promoting T1D.

## NK cells pro-angiogenic role in tumors and pregnancy

Angiogenesis is supported by transcription factor HIF-1α, which is induced under hypoxia to promote the expression of pro-angiogenic factors to stimulate blood vessel growth through the HIF-1α/VEGF axis.^348^ Hypoxia dramatically affects NK cells, as demonstrated in vitro and in cancer patients. One week after being exposed to hypoxia (1%O2), peripheral NK cells were enriched in CD56^bright^CD16^Neg^ phenotype and became capable of secreting VEGFA in the media that could increase HUVEC cell’s angiogenic capacity.^286^ A clear demonstration of the NK cell’s conversion to a pro-angiogenic phenotype was shown in renal cell cancer patients who had peripheral NK cells with a CD56^pos^ CD16^pos^ phenotype, but NK cells infiltrating renal cancer with a CD56^pos^ CD16^Neg^ phenotype, like dNK, with enrichment in genes of the hypoxia-inducible factor HIF-1α pathway.^349^ To understand the role of these NK cells in the tumors, it is important to learn from another conversion of NK cells to a pro-angiogenic phenotype observed in another normal physiological phenomenon, pregnancy. NK cells CD56^bright^CD16^Neg^, also called dNK, secrete an array of pro-angiogenic factors that regulate trophoblasts invasion and actively produce IL-8 and interferon-inducible protein-10 chemokines, CXCL10. dNK cells are anergic non-cytotoxic despite expressing NK activating receptors, including NKp44, NKp46, NKp30, and NKG2D.^350–352^ However, dNK cells express high levels of GNLY and are capable of killing virus infected stromal cells of the mother after activation,^353^ but do not kill the bacteria infected trophoblasts. Instead, they deliver GNLY to specifically kill the bacteria without harming the trophoblast^19^ or damaging the maternal-fetus interface.

dNK emerge from immature uterine NK cells originally from PB and which upon stimulation with IL-15, acquire KIRs and mature.^354^ The implantation of the embryo is an inflammatory process of the uterus primed by ovarian hormones to secrete IL-8, IL-15, IL-6, CXCL10, and CXCL11.^355^ These cytokines and chemokines attract decidual immune cells of which 70% are uterine NK cells. Survival of the embryo with its semiallogenic genetic stock in the uterus will depend on the tolerance of maternal immune cells. dNK cells at the maternal–fetal interface express inhibitory receptors such as KIR2DL1, KIR2DL2, L3, and Leukocyte immunoglobulin-like receptor subfamily B member 1 (LILRB1), which recognizes HLA-G to inhibit NK-cell cytotoxicity^132^ and inhibitory receptors CD94/NKG2A which interact with and HLA-E.^356^ Indeed, NKG2A genetic ablation in female mice caused suboptimal maternal vascular remodeling in pregnancy, reduced fetal weight, and abnormal brain development resembling the human syndrome pre-eclampsia.^357^ At the onset of pregnancy, the high expression of KIR2D in dNK and the upregulation of HLA-C in the stromal cells of the endometrium, which transform into decidua, are crucial. At the maternal-fetal interface, NK cells represent the majority of immune resident cells as they expand in uterus spiral arteries. Therefore, dNK cells have a productive role in pregnancy by regulating key developmental processes, including angiogenesis at the human fetal-maternal interface.^358^ dNK cells also appear to control oxygen levels by regulating uterine spiral artery development. Indeed, the absence of NK at the fetal-maternal interface increases hypoxia.^359^ Therefore, NK cells maintain an oxygen and nutrient-rich environment, influence trophoblasts, and promote the development of the invasive trophoblast lineage necessary for optimal blood supply between mother and fetus through the mother KIRs and fetal HLA interactions.^360,361^ Going back to tumor physiology, strikingly, the deletion of HIF-1α in NK cells reduced their recruitment into tumors, while it did not affect that of CD4 or CD8 T-cells. The lack of NK cell recruitment led to a reduction in tumor size through non-productive angiogenesis. This later is characterized by increased hypoxia and a high density of immature hemorrhagic blood vessels,^362^ suggesting that NK cells are required to mature blood vessels during the remodeling of tumor vasculature as in pregnancy. Krzywinska et al. showed that HIF-1α KO-NK cells prefer to reside in well-oxygenated areas, thus ignoring hypoxic regions that need their presence. Most importantly, HIF-1α was found to be required for the cytotoxicity of NK cells.^362^ The authors concluded that NK cells will balance excessive angiogenic tumor efforts by providing the angiostatic soluble VEGFR1 (sVEGFR1) to control VEGF bioavailability in an HIF-1α-dependent manner. While the role of HIF-1α in tumor angiogenesis is established in the above study and is in line with the events during pregnancy, the conclusions regarding NK cytolytic functions might depend on the tumor model used in the study. Another study showed that HIF-1α deletion unleashed NK cells cytolytic activity, but only against MHC I deficient tumors, and that this required IL-18.^363^ Single-cell analysis showed that HIF-1α inhibits IL-18 signaling, thus reducing NFkb signaling and IFNγ, reducing NK cell infiltration in tumors. Indeed, deletion of HIF-1α allows IL-18 secreted by myeloid cells to activate NK cells against MHC I deficient tumors. Of note, hypoxia also induces IL-18 to promote angiogenesis^364^ and it might be needed for the initial phase of gestation, but its upregulation in the decidua of patients was associated with recurrent miscarriages.^365^ IL-18 role in tumor hypoxia and pregnancy is complicated by its pleiotropic effect and its ability to induce more than 1000 genes in NK cells, as well as, the partial overlap with IL-2, IL-12, and IL-15 functions.^366^ Additionally, we have seen earlier that IL-18 and IL-12 can reverse the anergy of NK cells in MHC I deficient tumors,^114^ suggesting this cytokine is critical for NK-mediated immunotherapy. Another intriguing effect of IL-18 is its ability to convert CD56^dim^ to a helper CD56^bright^ CD16^Neg^ phenotype,^367^ which is potentially more pro-angiogenic. In summary, the presence of dNK cells at the maternal-fetal interface is driven by CD56^bright^ migration in response to cytokines and chemokines and probably hypoxia, sensed through HIF-1α. Interaction with trophoblasts triggers a pro-angiogenic dNK phenotype that helps in building spiral arteries, creates better and balanced oxygenation and brings more nutrients to the interface. Trophoblasts through HLA-E and HLA-G, represses dNK cytolytic activity and further promotes their pro-angiogenic role. Reduced dNK at the interface has been reported in pre-eclampsia,^368^ suggesting that the dNK to trophoblasts ratio is crucial for balanced angiogenesis. In this regard an intriguing question regarding the role of HIF-1α in initiating or maintaining this dynamic must be studied through the conditional knockout of HIF-1α, before and after pregnancy is established. Knockout of HIF-1α would prevent dNK cells migration to the interface. These investigations could confirm if HIF-1α KO dNK’s inability to correctly sense hypoxia is an important factor in pre-eclampsia or even parturition. The same animal model could also evaluate the impact of hypoxia sensing by NK cells in tumor initiation, metastasis, and angiogenesis. HLA-G found in trophoblasts of the placenta, plays a crucial role in maternal-fetal tolerance, acting as an immune checkpoint.^369^ Expression of HLA-F and HLA-G on migrating trophoblast support their invasion and interactions with uterine natural killer cells.^370^ HLA-G is also highly expressed in a variety of tumors and is involved in their immune escape, which is mediated by the interaction with immune cells, including NK cells.^371^ In tumors, HLA-G interacts with LILRB1/2 and KIR2DL4 to suppress cytotoxic T-cells and NK cells and promotes the expansion of immunosuppressive cells, Treg cells and MDSCs, creating an immunosuppressive microenvironment that aids tumor cells in evading the immune system. Moreover, KIR2DL4 expression is enhanced by IFNγ,^372^ suggesting a role in immune response regulation. Therefore, for a common purpose, KIR2DL4, by interacting with HLA-G, participates in pacifying the maternal-fetus interface and allows tumors to escape immunity.

## NK cells in senescence and developing cancers

Most established cancers have already escaped surveillance by immune cells, including NK cells. There is an emergent consensus around the decidualization of NK cells in the tumor microenvironment as in the maternal/fetal interface and even of some circulating NK cells in cancer patients, leading to anergy and even subservient status in tumors. The CD56^bright^, CD16^dim/neg^ NK cells could become pro-angiogenic, possibly hijacked and reprogrammed to benefit the tumor progression.^373,374^ Peripheral NK cells, which are mostly CD56^Dim^ CD16^positive^, are likely to intercept transiting metastatic cells. However, the less active CD56^Bright^CD16^dim/neg^ NK cells that localize in tissues are intrinsically less likely to achieve that. In a human of 73 kg, the total number of NK cells in the bone marrow where they are continuously produced is 4 × 10^9^. The blood and skin each harbor 2 × 10^9^, while a large majority (30%) of 5 × 10^9^ NK cells are found in the liver, and only 1 × 10^9^ can be found in the lymphatic system or the lungs.^375^ The most likely initial mechanism a developing cancer cell uses in the very initial stage would be the most potent inhibitory tool against NK cells, the MHC I complex. Interestingly, senescence, which shares many precursor states with tumorigenesis, such as accumulation of DNA damage or defective signaling and which is now proposed as an enabling hallmark of cancers,^376^ also leads to overexpression of MHC I.^377,378^ This could further inhibit the already subdued CD56^Bright^, CD16^dim/neg^ NK cells. Therefore, it stands to reason that because of the large population of senescent cells accumulating in aging tissues,^379,380^ there will be more inhibitory forces against NK cell populations. This is compounded in the elderly by the cross-the-board decline of immune cell functions that normally support NK cells by providing cytokines. Notably, macrophages’ reduction in numbers and bactericidal capacity,^381^ the decreased antigen presentation function in DC cells,^382^ the dwindling numbers of B-cells and their capacity to properly produce a diverse immunoglobulin repertoire,^383^ as well as the reduced stemness of hematopoietic stem cells, producing less lymphocytes such as T-cells.^384,385^ All these events may lead to reduced clearance of senescent cells and their accumulation in aging tissues and age-associated diseases.^386^ Senescent cells overexpress MHC I and their HLA-E expression consistently increases in aging human skin and melanocytic nevi compared to young skin. Blocking HLA-E interaction with ligand NKG2A on NK and CD8 T-cells allowed the killing of senescent cells by NK cells.^387^ A clear link between senescent and cancer cells was demonstrated by the reduction of spontaneous tumorigenesis and cancer-related death after the depletion of senescent cells in aging mice.^388^ An immediate question arises regarding why senescent cells accumulate in the elderly but not in the young. This could originate from the increased number of cells entering senescence in the elderly compared to the young. However, a study in mice showed that the expression of MHC I ligands and KIRs on NK cells also increases in the elderly,^389^ suggesting that NK cells also become less responsive to senescent cells. Another study in elderly humans showed a reduction of NKp30 and NKp46 expression in NK cells, suggesting reduced interactions with DCs and functions,^390^ with increased KIR expression in the CD56^bright^ population.^155^ However, the same study found evidence of some NK cells subset compensating for these deficiencies. For example, CD56^dim^ population increased and CD94 expression declined in the elderly in both NK subsets. Nonetheless, more evidence of reduced NK activity in the elderly is suggested by their reduced response to IL-2 and impaired cytokine signaling.^391^ It is plausible that senescence’s increased rate at older age is only due to the lack of immune cell reactivity, including from NK cells, leading to reduced clearance of senescent cells. This could lead, in turn to a critical mass of proinflammatory senescent cells with a SASP, which produce inflammatory cytokines like IL-1α/β, IL-6, IL-8, TNFα, chemokines, DNA, microRNAs, proteases such as matrix metalloproteinases, wound healing factors PDGF-AA, endothelial vascular factor VEGF and senescence promoting factor IGFBP4/7,^392–395^ extracellular vesicles and exosomes containing cytokines such IL-15^396^ or Heat shock proteins.^397^ Additionally, SASP from senescent cells can induce the senescence of neighboring cells,^398^ leading to a vicious cycle of senescent cell accumulation. However, this conversion could also transform neighboring cancer cells into senescent non-replicating cells.^399^ This effect is thought to be protective, reducing cancer and providing an evolutionary explanation of the benefit of senescent cells. However, as mentioned earlier, depleting senescent cells in animal models reduced cancer frequency.^388^ Senescent cells overexpress decoy receptor 2, allowing them to escape the FasL death pathway. The mechanism through which NK cells remove senescent cells involves granular exocytosis, mostly through overexpressed ligands MICA/B, ULBP1-3, PVR, and nectin-2 binding to activating receptors NKG2D and DNAM-1.^400–402^ Additionally, senescent cells in aging tissues secrete SASP with chemokines GM-CSF, CCL2, 3, 4, and 5, CXCL1, 9, 10, and 11, which attract immune cells, including NK cells.

## Crosstalk with other immune cells

NK cells increase inflammation after activation by tumor and virus-infected cells by secreting inflammatory cytokines such as IFNγ,^22,23^ which activates macrophages and neutrophils,^24^ T-cells,^25^ and B-cells.^26^ However, cancer cells treated with IFNγ become resistant to NK cells, suggesting that NK cells secretion of IFNγ may be designed to involve other immune cells^27^ to remedy their deficiency, suggesting a redundancy mechanism.

### Macrophages

**Fig. 7:**
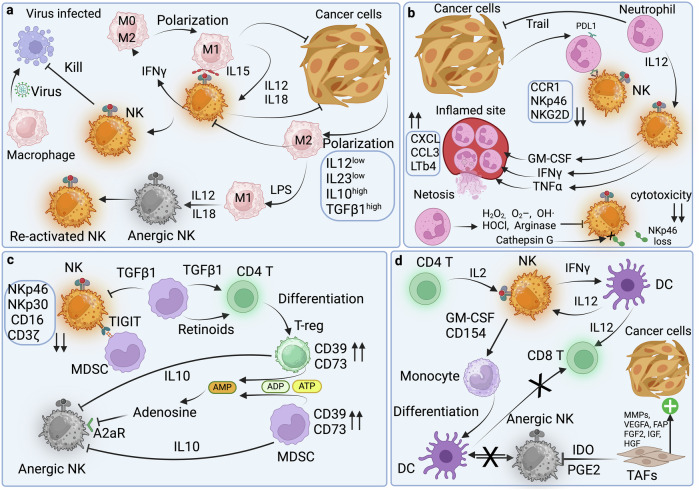
Crosstalk with other immune cells. **a** NK cells secreted IFNγ can help polarize macrophages (M0, M2) to antitumor M1 phenotype. Macrophages reciprocate by IL-12 and IL-15 trans-presentation to increase IFNγ production by NK. Virus-infected Macrophages are killed by NK cells, and anergic NK cells may be reactivated by macrophages IL-12 and IL-18. **b** Neutrophils enhance tumor defense by triggering TRAIL-mediated apoptosis. They release IL-12 to boost IFNγ and perforin in NK cells but also downregulate NK cell receptors via PD-L1 upregulation induced by G-CSF. To support neutrophil function, NK cells reciprocate by secreting IFNγ, GM-CSF, and TNFα. However, neutrophils can inhibit NK cells through NET-mediated NKp46 cleavage, while tumor-associated neutrophils suppress immune responses via ARG1 release and ROS production. **c** MDSCs and Tregs suppress NK cell function through direct contact or secretion of TGFβ1 and IL-2 depletion, alongside IL-10 production. MDSCs also employ TIGIT to inhibit NK cells, reducing CD3ζ and impairing NK cell receptors. They can also hinder NK cells through direct interaction with NKp30. However, MDSCs can induce IFNγ release in NK cells via NKG2D activation by RAE-1 ligand. MDSCs promote the trans-differentiation of naive CD4^+^ T cells into Foxp3^+^ Tregs. Additionally, MDSCs and Tregs convert extracellular ATP and ADP to cAMP and adenosine by CD39 and CD73, inhibiting NK cell antitumor responses via A2AR binding. **d** T-cell production of IL-2 activates NK cells, which, by the production of IFNγ, activates DCs. DCs reciprocate by IL-12 to reinforce IFNγ production and stimulate CD8 T-cells. NK cells producing GM-CSF and CD154 can induce CD14^+^ monocyte differentiation into DCs. TAF production of PGE2 and IDO can exhaust NK cells, thereby blocking their mutual activation with DCs and subsequent CD8 T-cell activation

Macrophages derive from circulating monocytes and reside in tissues where they adopt different phenotypes, such as unpolarized M0 and two polarized states (M1), which is anti-tumor and pro-inflammatory, and (M2) which is pro-tumor and anti-inflammatory. Due to their abundance in tissues, they are most likely the first to discover sites of infections by viruses, bacteria, and parasites. Overactivated or infected macrophages will be killed by bystander-activated NK cells.^83^ Initially, NK cells, in direct contact with these macrophages, increase their degranulation marker CD69 and IFNγ expression^403^ and collaborate closely with macrophages to control the infection and inflammation (Fig. 7a). NK cells and IFNγ are required and sufficient for the polarization of tumor-associated macrophages (TAMs) to M1, which protect against tumor growth even in the absence of adaptive immunity.^404^ Depending on the infectious agent, NK cells will express specific receptors to specific ligands, the involved macrophages express. Macrophages stimulated or infected by cytomegalovirus will stimulate co-activating receptors 2B4, NKp46, and DNAM-1 on NK cells.^405,406^ At the same time, stimulation by Streptococcus pneumonia induces clearance through activation of NKp46^407^ and stimulation by bacterial moieties such as lipopolysaccharides (LPS), an outer membrane component of gram-negative bacteria, as well as Mycobacterium tuberculosis, Sendai or Influenza A virus,^408^ induce the ligands, retinoic acid early inducible-1 (RAE-1),^409^ ULBP1-3,^405^ MICA, and MICB,^410^ which will activate the NKG2D receptor. The developing tumor microenvironment, with its increased inflammation, acidic metabolism, hypoxia, and chemokines, attracts monocytes to seed the tumor with what is to become TAMs. Monocyte chemoattractant protein CCL2 (MCP-1), which interacts with CCR2, plays a prominent role in this recruitment.^305^ Recruited monocytes are either polarized into M1 macrophages characterized by IL-12^high^ IL-23^high^ IL-10^low^ and have phagocytic and antitumor activity, or M2, which are IL-12^low^ IL-23^low^ IL-10^high^ and TGFβ1^high^ with no phagocytic activity and secrete TGFβ1 to inhibit NK cells anti-tumor activity.^411^ However, these two states are interchangeable, depending on the balance between immunosuppression and immunostimulation.^412^ For example, stimulation by LPS can revert M0 and M2 macrophages to an M1 phenotype, leading to NK cell activation.^83^ This activation could help restore anergic NK activity by cytokines such as IL-18 and IL-12.^114^ Similarly, IL-15 trans presented by M1 macrophages after contact with bacterial moieties leads to strong NK cell activation.^413^ Virus-infected Macrophages are killed by NK cells. In patients with severe COVID-19, a surge in many proinflammatory cytokines leads to acute respiratory disease syndrome originating from macrophage-activation syndrome. In these patients, the number of NK cells was dramatically reduced, and their activation by K562 leukemia was impaired compared to healthy controls. Additionally, these patients had very low levels of IL-12, IL-15, and IL-21 needed to activate NK cells. These findings suggest that in severe COVID-19 patients, NK cells are highly exhausted and fail to kill virus-infected macrophages that produce proinflammatory cytokines.^91^ To evaluate the efficacy of engineered allogenic cord blood NK cells, clinical trial NCT04324996 (Table 1) is evaluating NKG2D-ACE2 CAR-NK targeting the S protein of SARS-CoV-2 and NKG2DL on the surface of infected cells with ACE2 and NKG2D, respectively.

**Table 1:** List of clinical trials using CAR-NK, TCR-NK, and BICAR-NK

Target	Trial identifier	NK source	Disease	Phase	Study Status	First Posted
Clinical trials using CAR-engineered NK cell therapy in malignant tumors						
CD5	NCT05110742	Cord blood	R/R hematological malignancy	I/II	Recruiting	2021
CD7	NCT02742727	NK-92	CD7 + R/R leukemia and lymphoma	I/II	Unknown	2016
5T4	NCT05194709	Not disclosed	Advanced solid tumors	I	Recruiting	2022
BCMA	NCT05182073	iPSC	MM	I	Recruiting	2022
	NCT06242249	Not disclosed	R/R MM	I/II	Not yet recruiting	2024
	NCT05008536	Cord blood	R/R MM	I	Unknown	2021
	NCT05652530	Healthy donor	R/R MM	I	Recruiting	2022
	NCT03940833	NK-92	R/R MM	I/II	Unknown	2019
	NCT06045091	Allogenic NK	R/R MM/ Plasma cell leukemia	I	Recruiting	2023
CD123	NCT06006403	Not disclosed	AML/blastic plasmacytoid dendritic cell neoplasm/relapse leukemia or leukemia	I/II	Recruiting	2023
	NCT05574608	Allogenic NK	R/R AML	I	Recruiting	2023
	NCT06201247	Healthy donor	R/R AML	I	Recruiting	2024
CD19	NCT05020678	PB-NK	B-cell malignancies	I	Recruiting	2021
	NCT05654038	Allogenic NK	B-Cell LL/Lymphoma	I/II	Recruiting	2022
	NCT06334991	NK-92	R/R NHL	I	Not yet recruiting	2024
	NCT04887012	Haploidentical donor	R/R B-cell NHL	I	Unknown	2021
	NCT01974479	Haploidentical donor	B-cell ALL	I	Suspended	2013
	NCT05336409	iPSC	R/R CD19 + B-Cell malignancies	I	Recruiting	2022
	NCT05739227	Allogenic NK	ALL/B-cell lymphoma/CLL	I	Recruiting	2023
	NCT04639739	Not disclosed	R/R B-cell NHL	I	Unknown	2020
	NCT05673447	Allogenic NK	Diffuse large B cell lymphoma	I	Recruiting	2023
	NCT05472558	Cord blood	B-cell NHL	I	Recruiting	2022
	NCT04887012	Haploidentical donor	B-cell NHL	I	Unknown	2021
	NCT05336409	iPSC	R/R CD19 + B-Cell malignancies/NHL	I	Recruiting	2022
	NCT05563545	Not disclosed	ALL	I	Completed	2022
	NCT05410041	Not disclosed	ALL/CLL/NHL	I	Unknown	2022
	NCT05645601	Allogenic NK	R/R B-cell hematologic malignancies	I	Recruiting	2022
	NCT06464861	Cord blood	R/R B cell lymphoma	I	Not yet recruiting	2024
	NCT03056339	Cord blood	B-lymphoid malignancies	I/II	Completed: Phase I Interim results reported 2020^90^ Phase 1/2 results reported 2024^579^	2017
	NCT05618925	NK-92	R/R NHL	I	Recruiting	2022
	NCT04796675	Cord blood	B-cell lymphoid malignancies	I	Unknown	2021
	NCT04796688	Not disclosed	ALL/CLL and B-cell lymphoma	I	Recruiting	2021
	NCT05379647	Not disclosed	R/R B-cell ALL	I	Recruiting	2022
	NCT03690310	iPSC	R/R B-Cell lymphoma	I	Recruiting	2018
	NCT03824951	iPSC	R/R B-Cell lymphoma	I	Recruiting	2019
	NCT01974479	Haploidentical donor	B-cell ALL	I	Suspended	2013
	NCT00995137	Haploidentical donor	R/R ALL	I	Completed	2009
	NCT06206902	Not disclosed	NHL	I	Recruiting	2024
	NCT02892695	NK-92	CD19+ leukemia/ lymphoma	I/II	Unknown	2016
	NCT05020015	Not disclosed	R/R B-cell NHL	II	Not yet recruiting	2021
	NCT04245722	iPSC	R/R B-NHL/CLL	I	Recruiting Interim trial results 2021^583^	2020
CD22	NCT03692767	iPSC	R/R B-Cell lymphoma	I	Unknown	2018
CD33	NCT05008575	Not disclosed	AML	I	Unknown	2021
	NCT02944162	NK-92	AML	I/II	Unknown	2016
	NCT05665075	Allogeneic NK	AML	I	Recruiting	2022
CD70	NCT05092451	Cord blood	R/R Hematological malignances	I/II	Recruiting	2021
	NCT05703854	Cord blood	Renal cancer/ mesothelioma/osteosarcoma	I/II	Recruiting	2023
Claudin18.2	NCT06464965	Cord blood	Gastric cancer/pancreas adenocarcinoma	I	Not yet recruiting	2024
Claudin6	NCT05410717	Autologous PB-NK	CLDN6+ advanced solid tumors	I	Recruiting	2022
CLL1	NCT06307054	Patient or healthy donor	R/R AML	I	Recruiting	2024
	NCT06027853	iPSC	AML	I	Recruiting	2023
DLL3	NCT05507593	Not disclosed	Extensive stage SCLC	I	Recruiting	2022
HER-2	NCT04319757	Not disclosed	Advanced or metastatic HER2+ solid tumors	I	Recruiting	2020
	NCT03383978	NK-92	Recurrent HER2+ glioblastoma	I	Recruiting	2017
Mesothelin	ChiCTR2100048100	Autologous	Refractory epithelial ovarian carcinoma	0	Recruiting	2021
	NCT03692637	iPSC	Epithelial ovarian cancer	I	Unknown	2018
MUC1	NCT02839954	Not disclosed	R/R MUC1+ solid tumors	I/II	Unknown	2016
NKG2D ligands	NCT05528341	NK-92	R/R solid tumors	I	Recruiting	2022
	NCT05247957	Cord blood	R/R AML	NA	Terminated	2022
	NCT03415100	PB-NK	Metastic solid tumors	I	Unknown Interim results reported 2019^584^	2018
	NCT06478459	Not disclosed	Non-resectable pancreatic cancer	I	Not yet recruiting	2024
	NCT05776355	Not disclosed	Platinum-resistant recurrent ovarian cancer	NA	Recruiting	2023
	NCT05213195	Not disclosed	Refractory metastatic colorectal cancer	I	Recruiting	2022
	NCT06379451	Not disclosed	MM	I	Not yet recruiting	2024
	NCT04623944	Allogeneic NK	Refractory MDS and AML	I	Not yet recruiting	2020
	NCT05734898	Not disclosed	R/R AML	NA	Recruiting	2023
PSMA	NCT03692663	iPSC	Castration-resistant prostate cancer	I	Not yet recruiting	2018
TAA (Not disclosed)	NCT05856643	Not disclosed	Ovarian epithelial carcinoma	I	Not yet recruiting	2023
	NCT05686720	Not disclosed	Advanced triple negative breast cancer	I	Not yet recruiting	2023
	NCT05845502	Not disclosed	Advanced hepatocellular carcinoma	NA	Not yet recruiting	2023
TROP2	NCT06454890	Not disclosed	NSCLC	I/II	Not yet recruiting	2024
	NCT06358430	Cord blood	Colorectal cancer/MRD	I	Not yet recruiting	2024
	NCT05922930	Cord blood	Pancreatic cancer/ovarian cancer/adenocarcinoma	I/II	Recruiting	2023
	NCT06066424	Cord blood	Solid tumors	I	Recruiting	2023
PD-L1	NCT04847466	NK-92	Gastroesophageal Junction (GEJ) Cancers/advanced HNSCC	II	Recruiting	2021
	NCT06239220	NK-92	Recurrent and metastatic HNSCC	II	Recruiting	2024
	NCT04390399	NK-92	Pancreatic cancer	II	Recruiting	2020
ROBO1	NCT03940820	Not disclosed	Solid tumor	I/II	Unknown	2019
CD19/CD22	NCT03824964	iPSC	Refractory B-Cell Lymphoma	I	Unknown	2019
CD33/CLL1	NCT05215015	Not disclosed	AML	I	Unknown	2022
	ChiCTR2100047084	Not disclosed	R/R AML	I	Recruiting	2021
	NCT05987696	iPSC	AML/MRD	I	Not yet recruiting	2023
CD33/DLL3	NCT06367673	iPSC	AML	I	Recruiting	2024
CD19/70	NCT05667155	Cord blood	R/R B-cell NHL	I	Recruiting	2022
	NCT05842707	Cord blood	R/R B-cell NHL	I/II	Recruiting	2023
CD33/TIM3	ChiCTR2100043081	Cord blood	AML	0	Recruiting	2021
MICA/B	NCT06342986	iPSC	Gynecologic cancer/ovarian cancer/fallopian tube cancer/primary peritoneal cavity cancer	I	Not yet recruiting	2024
CLL1	NCT06027853	iPSC	AML	I	Not yet recruiting	2023
Clinical trials using TCR-engineered NK cell therapy in malignant tumors						
PRAME	NCT06383572	Cord blood	Myeloid malignancies	I/II	Recruiting	2024
NY-ESO-1	NCT06083883	Healthy donor	Synovialsarcoma/ myxoid/round cell Liposarcoma	I/II	Suspended	2023
Clinical trials using Bi-CAR-engineered NK cell therapy in malignant tumors						
CD33/FLT3	NCT06325748	Healthy donor	AML/MDS/CD33+and or FLT3+ Hematological Malignancies	I	Recruiting	2024
CD30/CD16A	NCT04074746	Cord blood	R/R CD30 + HL and NHL	I/II	Not yet recruiting	2019
ROBO1	NCT03941457	Not disclosed	Pancreatic cancer	I/II	Recruiting	2019
ROBO1	NCT03931720	Not disclosed	Malignant Tumor	I/II	Recruiting	2019
Clinical trials using CAR-engineered NK cell therapy in Autoimmune Diseases or COVID19						
CD19	NCT06464679	Not disclosed	Autoimmune diseases	I	Not yet recruiting	2024
	NCT06318533	Not disclosed	Autoimmune diseases	I	Recruiting	2024
	NCT06208280	Not disclosed	Autoimmune diseases	I	Recruiting	2024
	NCT06468683	Not disclosed	Lupus erythematosis	I	Not yet recruiting	2024
	NCT06377228	Not disclosed	Refractory lupus nephritis	I	Not yet recruiting	2024
	NCT06421701	Not disclosed	SLE	I	Not yet recruiting	2024
	NCT06255028	Not disclosed	SLE	I	Not yet recruiting	2024
	NCT06010472	Not disclosed	SLE	I	Recruiting	2023
	NCT06337474	Not disclosed	Thrombocytopenia Alloimmune	I	Not yet recruiting	2024
	NCT06469190	Not disclosed	R/R Immune Nephropathy	I	Not yet recruiting	2024
NKG2D ligands	NCT04324996	Cord blood	COVID-19	I/II	Unknown	2020

*ALL* acute lymphoblastic leukemia, *AML* acute myeloid leukemia, *BCMA* B cell maturation antigen, *CAR* chimeric antigen receptor, *CLL* chronic lymphocytic leukemia, *CR* complete remission, *CRS* cytokine-release syndrome, *HLA* human leukocyte antigen, *hnCD16* high-affinity non-cleavable CD16, *HNSCC* head and neck squamous cell carcinoma, *iPSC* induced pluripotent stem cell, *MDS* myelodysplastic syndrome, *MRD* minimal residual disease, *MICA/B* MHC class I chain-related protein A and B, *NHL* non-Hodgkin lymphoma, *NK* natural killer, *NSCLC* non-small cell lung cancer, *ORR* objective response rate, *PB* peripheral blood, *PSMA* prostate specific membrane antigen, *ROBO1* roundabout homolog 1, *R/R* relapsed or refractory, *SCLC* small cell lung cancer, *SLE* systemic lupus erythematosus, *5T4* oncofetal trophoblast glycoprotein, *TCR* T-cell receptor, *COVID-19* Coronavirus disease 2019, *TIM3* T-cell immunoglobulin and mucin domain 3

### Neutrophils

Neutrophils are required for NK cell development in mice and humans,^414^ and patients with chronic neutropenia have increased frequencies of CD56^bright^ NK cells and lack mature CD56^dim^ NK cells.^415^ Neutrophils have an anti-tumor effect mediated by TNF-related apoptosis-inducing ligand (TRAIL), which can induce apoptosis in leukemic cells^416^ (Fig. 7b). Additionally, neutrophils release IL-12, crucial for NK cells’ enhanced IFNγ and perforin production.^417^ However, in tumor-bearing animals, neutrophils downregulated chemokine receptor CCR1, NKp46, and NKG2D expression in NK cells through direct contact with NK cells, weakening their tumor infiltration and responsiveness.^418^ This immunosuppression was mediated by neutrophils’ increased PD-L1 expression, induced by G-CSF, the regulator of neutrophils’ generation and differentiation, and the STAT3 signaling. Since NK cells also produce pro-inflammatory cytokine GM-CSF, they might attract neutrophils, thereby upregulating pro-inflammatory CXCL2, CCL3, and LTb4, which sustain immune cell recruitment into inflamed tissues.^334^ It is unclear whether neutrophils have a beneficial role in NK cell’s antitumor activity. Still, the fact that their numbers are increased in cancer patients^304^ and that neutrophils are a critical component of the inflammatory process, which is now accepted as part of tumorigenesis,^419^ suggests that neutrophils may be mostly immunosuppressive forces in tumors, promoting angiogenesis, extracellular matrix remodeling, metastasis, and immunosuppression.^420^ By secretion of IFNγ, GM-CSF, and TNFα, NK cells can enhance neutrophil survival, activation,^421,422^ and the formation of Neutrophils Extracellular Traps (NET).^423^ However, neutrophils can inhibit NK cells through NKp46 cleavage by NETs enriched in cathepsin G.^424^ Tumor Neutrophils are the primary source of Arginase I (ARGI),^425^ which they store in granules. ARG1 depletion of L-Arginine by hydrolysis to L-ornithine and urea profoundly suppresses T-cell immune responses.^426^ Finally, neutrophils produce reactive oxygen species (ROS) such as H_2_O_2_, O_2_^–^, OH·, and HOCl, which reduce NK survival and cytotoxicity.^427^ Therefore, NK cells and neutrophils can modulate each other.

### Myeloid-derived stem cells (MDSCs) and Tregs

These cells mediate NK cell function suppression by direct contact or secretion of TGFβ1^428–430^ and IL-2 depletion,^431^ respectively. However, both produce immunosuppressive IL-10. MDSCs could also suppress NK cells via the inhibitory receptor TIGIT (Fig. 7c), an effect abrogated by TIGIT blockade.^432^ Additionally, NK exposed to MDSCs have reduced CD3ζ, with impaired natural cytotoxicity receptors NKp46, NKp30, and CD16.^433^ MDSCs can also inhibit NK cells by direct interaction with NKp30.^434^ However, MDSCs were reported to stimulate NK cells to release IFN-γ by activating NKG2D by MDSCs ligand RAE-1.^435^ Retinoids and TGFβ produced by MDSC promote the trans-differentiation of naive CD4^+^ T cells into Foxp3^+^ Tregs.^436^ Interestingly, MDSCs and Tregs can convert extracellular ATP and ADP in the TME to cAMP by CD39 and, subsequently, CD73 dephosphorylates AMP to adenosine, which by binding to adenosine receptor (A2AR) on NK cells inhibits their antitumor response.^437^

### Collaboration, suppression, and murder of T-cells

IL-2 released by activated T cells plays a role in NK cell activation and IFNγ production.^438,439^ Conversely, T helper cell type 1 (Th1) polarization requires IFNγ provided by activated NK cells.^440^ The same IFN-γ secreted by NK cells will also stimulate IL-12 production by DCs, which activates CD8 + T anti-tumor activity^441^ (Fig. 7d). Similarly, activation of DCs by Cetuximab-activated NK cells enhanced antigen-specific T-cell immune responses in patients with head and neck cancer.^442^ In another mutual collaboration, NK cells expressing the OX40 ligand and B7 will induce the proliferation of T-cells.^443^ Therefore, the presence of both NK and T-cells in tumors will be synergistic and beneficial, as shown in colorectal cancer patients where NK cells and CD8 + T cell infiltration is associated with prolonged patient survival.^444^ NK cells ability to constitutively secrete TGFβ1^28^ may reduce inflammation and inhibit T-cell cytotoxicity and proliferation,^29^ allowing tissue repair.^30^ Additionally, NK cells secretion of immunosuppressive IL-10 in early response to systemic but not to local infection,^32,33^ indirectly limits T-cell activation by blocking DCs secretion of IL-12 and production of factors involved in antigen presentation^34^ and T-cell anti-viral response,^35^ thus promoting T-cell exhaustion^36^ and reducing immune-mediated damage to tissues. The involvement of NK cells in directly dampening T-cell activity by cytokines (IL-10, TGFβ1) and indirectly by blocking IL-12 cytokine secretion by DCs through NKp30 has now been extended to the direct kill of activated T-cells that express B7-H6.^95^ This finding also applies to CAR-CD19-T cells, which, upon knockout of their B7-H6, escape being killed and expand more. Concurrently to B7-H6-induced expression on activated T-cells, Kilian et al, observed downregulation of HLA-E and C-type lectin domain family 2 member D, perhaps further enhancing T-cells killing by NK cells. It is interesting to note that NK cells also kill immature DCs (see below) through NKp30 recognition and that this kill is prevented in mature DCs by enhanced expression of HLA-E.

### Dendritic cells

NK cells enhance DCs maturation, IL-12 production, and priming of CD4(+) T-cell proliferation and IFNγ secretion^445^ (Fig. 7d). Immature DCs are killed by a subset of NK cells lacking KIRs^446^ and through signals mediated by NKp30,^76^ whereas mature DCs are protected from NK lysis by upregulation of MHC I molecules,^447^ HLA-E in particular.^446^ This DCs selection is important for the downstream development of adaptive immunity. CD56^bright^ NK cells producing GM-CSF and CD154 can induce CD14^+^ monocyte differentiation into DCs, in RA and psoriatic arthritis patients.^330^ Therefore, NK cells promote monocyte differentiation into DC to sustain the disease. NK cells expression of ChemR23^313^ and CCRL2,^314^ which by attraction to chemerin recruit NK to colocalize with Chem23-expressing DCs in inflammatory sites. ChemR23 is also expressed on macrophages, adipocytes, and endothelial cells,^315–317^ suggesting they all colocalize with NK cells. As mentioned earlier, human NK cells activated by IL-2 express SIPR1,4 and 5, a G-coupled receptor protein chemoattracted to bioactive lipid S1P.^318,319^ Receptor SIPR5 expression by NK and DCs suggests their colocalization.^320^ Similarly, the induction of IFNγ from CD56^bright^ subset interaction with DCs induces surface molecules CD62L, CCR7, and CXCR3^331^ in NK cells, thus increasing their potential to colocalize with DCs.

### Tumor-associated fibroblasts (TAFs)

TAFs are heterogeneous populations derived from various cell types, including normal fibroblasts, smooth muscle cells, pericytes, and tumor epithelial cells transformed by the epithelial-mesenchymal transition. This heterogeneity creates a complex matrix in the tumor environment mainly focused on tissue remodeling by producing MMPs, VEGFA, and FAP. It also produces tumor-promoting factors, including FGF2, IGF, and HGF, and immunosuppressive factors TGFβ, PGE2, and IDO, as well as factors promoting inflammation, like chemokines CCL2, CXC, CXCL12, CXCL8, and IL-6. The concept that tumor-promoting inflammation by cancer cells and by the associated tumor microenvironment can support cancer progression is a well-established hallmark of cancer.^376^ TAFs production of inflammatory mediators Prostaglandin E2 (PGE2) and Indoleamine 2,3-dioxygenase (IDO) can suppress NK cells.^448^ PGE2, a significant product of cyclooxygenases, suppresses NK cell function by signaling through PGE2 receptors Ep(1-4),^449–451^ with Ep4 being the most potent at inhibiting IFNγ production.^452^ Additionally, tumor-derived PGE2 signaling through EP2 and EP4 receptors increases T-reg cell activity in lung cancer,^453^ further antagonizing NK cells. Similarly, PGE2 impairs the NK cell and DCs interactions, reducing IL-12 secretion by DCs and CD4 T-cell polarization.^454^ Tumor PGE2 was also reported to inhibit chemokine receptors on cDC cells, preventing their attraction by CCL5 and XCL1 secreted by NK cells.^322^ IDO metabolizes Tryptophan to L-kynurenine, which inhibits the upregulation of NKp46 and NKG2D under IL-2 stimulation. Therefore, IDO depletes tryptophan and starves, particularly T-cells,^455^ thus disrupting the cooperation between NK and T-cells, inhibiting CD4 and CD8 T-cells,^456^ and NK cell cytotoxicity.^457^ Knockdown of IDO in cancer cells enhanced their sensitivity to NK cells in vitro and promoted their accumulation in the tumors.^458^

### Platelets

NK cells are essential for controlling metastasis. However, this task might be impeded by platelets, which are small non-nucleated fragments of megakaryocytes that aggregate with fibrin deposits on cancer cells’ surface in a process miming coagulation.^459^ Additionally, aggregated platelets could transfer MHC I to MHC I-deficient cancer cells, thereby interfering with the missing self-recognition by NK cells.^460^ In addition to the physical shielding of cancer cells, platelets are the richest source of TGFβ1, downregulating NKG2D in NK cells.^461^

## Natural killer cells mediated cancer immunotherapies

Currently, NK cells used therapeutically are derived from PB,^462^ umbilical Cord blood (CB), and in vitro differentiated CD34^+^ progenitor cells,^463^ induced pluripotent stem cells (iPSCs),^464^ and immortalized NK cell lines, most notably NK-92 cell line which lacks most KIRs and is more likely to resist inhibition.^465^ NK cells isolated from PB are, by definition, mature with a complete armamentarium. However, they are stubbornly challenging to engineer, especially if repeated manipulations are needed to build on previous improvements. Like other continuously produced innate immune cells, primary NK cells lifespan is short (~2 weeks). This poses serious challenges for their use in immunotherapies. By definition, NK cells derived from CB are allogenic and were shown to induce monocyte-to-dendritic cell conversion in patient-derived cultures of primary and metastatic colorectal cancer.^466^ CB NK cells proliferate better than adult PB NK cells^467^ and can be obtained without the screening and leukapheresis required for adult PB NK. PB and CB NK cells can be expanded to large numbers using antigen-presenting feeder cells.^468,469^ Ex-vivo-activated autologous NK cells display less anti-tumor efficacy^470^ than allogeneic NK cells^471^ because self-class I HLA signaling suppresses NK cytotoxicity and cytokine release.^472^ Additionally, unlike allogeneic T-cells, allogeneic NK cells mediate anticancer effects without causing graft versus host disease.^473–475^ However, the effectiveness of allogeneic donor NK stimulated ex-vivo is reduced by competition for cytokines^79,476^ and approaches relying only on CAR technology, as CAR-NK cells suffer from tumor cell escape by HLA aberrant expression and epitope loss.^477^ Moreover, cytokine administration would be required to sustain NK cells after in vivo transfer,^478^ exposing patients to potential side effects. Therefore, developing novel NK cell activation strategies to reduce cytokine toxicity, increase resistance to immunosuppression, and enhance NK cell persistence is critical.

### Arming NK cells with activating cytokine signaling

The activating signaling from cytokines serves a different purpose than the signaling from activating ligand/receptor interactions. Ligand/receptor activation signals mobilize the machinery for cell killing but can also trigger proliferation. On the other hand, cytokines signaling initially directs the NK cell’s maturation and later serves to enhance their survival and proliferation. This is achieved mainly through transcription factors STATs, which produce a battery of genes that will maintain the NK cellular homeostasis. Ligand-receptor activation signals mostly originate from aberrant cells, while cytokines are secreted and used mainly between immune cells. NK cells do not manufacture interleukins and depend on other immune cells for survival. NK cells will respond to IL-2 and IFNγ from T cells, IL-12, IL-15, IL-18, and IL-21 from DCs and macrophages. IL-2 stimulates both NK and T cells, including Tregs. For this reason, and to reduce IL-2 toxicity in immunotherapies, efforts were devoted to creating potent IL-2 forms that discriminate between NK cells and Tregs.^479–481^ We contributed to this effort by tethering IL-2 to its receptor IL2Rβ.^288^ Our strategy abrogated IL-2 toxicity and allowed enhanced NK cell activation and cytotoxicity in vitro and in vivo. IL-15 is the only cytokine capable of inducing NK cell proliferation in vivo. Embattled T-cells receive survival factors and cytokines in the tumor as trans-presented IL-15 by DCs.^482^ Surprisingly, the mature NK population could collapse in vivo when DCs are depleted, suggesting that most NK stimulation in vivo occurs through IL-15 trans presentation by DCs.^483^ IL-15 is among a few cytokines that can extend telomeres by enhancing telomerase activity in NK, NKT, and CD8 T-cells.^484^ However, telomeres erode at a rate of 50 bp/year in human T-cells,^485^ with old individuals having shorter telomeres than young subjects.^486^ This implies that differentiated primary NK cells used in immunotherapies will probably have similar shortcomings. In vitro, the viability of CB-NK expressing soluble IL-15 and CAR-CD19 declined precipitously from day four post-plating,^487^ suggesting telomeres loss due to insufficient activation from CARs. IL-15 substantially improved CB-NK use in NK cells, especially when combined with the knockout of the CIS gene.^488^ However, reports suggest that secreted IL-15 expands primary and CB-NK cells but causes severe^487^ to lethal toxicity and cytokine release syndrome in animal models.^489^ Others reported that NK cell chronic stimulation by IL-15 leads to exhaustion by a metabolic defect.^490^ IL-12 produced by DCs and macrophages stimulates NK cells and leads to IFNγ production, which enhances DCs activation and induces T-cell polarization. Additionally, we have seen earlier that IL-12 with IL-18 can reverse the anergy of NK cells in MHC I deficient tumors,^114^ suggesting IL-12 will have a critical role in NK-mediated immunotherapy. However, the use of IL-12 in the clinic is hampered by its induced neutropenia and thrombocytopenia.^491^

The generation of mouse cytomegalovirus-specific long-lived memory NK cells with higher responses compared to naïve NK cells was shown to be dependent on IL-12-STAT4 signaling.^492^ Short-term pre-activation with a combination of IL-12/15/18 can induce memory characteristics in human NK cells.^462^ These memory-like NK cells have prolonged expression of CD25, capable of responding to IL-2 at picomolar concentrations.^493^ Therefore, strategies to develop memory NK cells ex vivo for clinical therapy are worthy of investigation.

IL-18, when combined with IL-12, potentiates the production of IFNγ and TNF in NK cells.^494^ Alone, IL-18 induces NK cells with a helper phenotype expressing chemokine CCR7 that migrate to secondary lymphoid organs,^367^ where they could potentially synergize with adaptive immunity. However, IL-18 pleiotropic effect, role in tumor hypoxia and pregnancy, and its ability to induce more than 1000 genes in NK cells, as well as its overlap with IL-2, IL-12, and IL-15 functions^366^ render its use in immunotherapy problematic. Another intriguing effect of IL-18 is its ability to convert CD56^dim^ to helper CD56^bright^ CD16 ^Neg^ phenotype,^367^ which is potentially more pro-angiogenic. IL-18 is normally inactivated by binding to serum IL-18 binding protein. However, a remarkable recent advance was able to circumvent this hurdle.^495^

IL-21 induces the transcription of many genes,^496^ including suppressors of cytokine signaling, Socs1, and Socs3, which downregulate the JAK–STAT pathway and inhibit IL-2 signaling.^497,498^ IL-21 activates Stat3,^499,500^ and Stat1.^501^ This latter leads to IFNγ production.^502^ IL-21 showed some benefit when used as monotherapy in the clinic but will probably need to be combined with other modalities.^503^ IL-21 is a B-cell growth factor that can potentially promote the growth of lymphomas.^504^ Therefore, its use as a soluble factor entails some risks. Acknowledging that strategies using single cytokines are less likely to succeed is also important. Instead, a rationale for efficient combinations of cytokines such as IL-2 or IL-15 with IL-21, IL-12, or IL-18 should be developed. For example, IL-15 may preserve telomeres better than IL-2. IL-21 may increase the metabolic fitness of NK cells, while IL-12 and IL-18 may reverse exhaustion. However, the best combination of cytokines may still require their use with other modalities.

### Countering Immunosuppressive factors

**Fig. 8:**
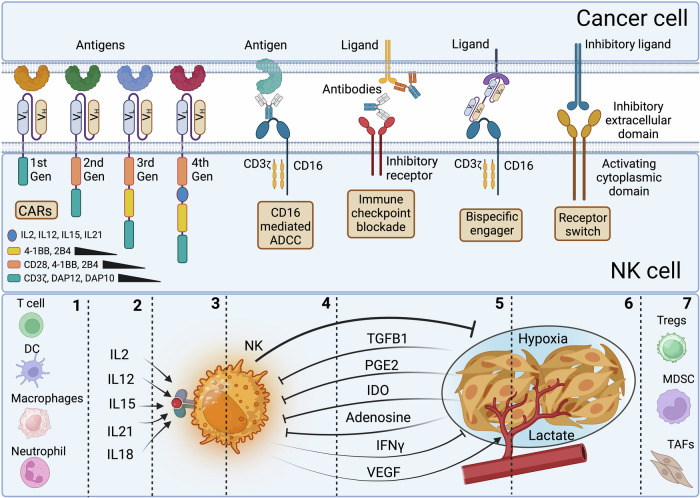
Strategies for arming and deploying NK cells. Chimeric antigen receptor (CAR) comprises an extracellular domain with the single-chain variable fragment scFv region (H heavy and L light chain) connected by a flexible hinge region mainly derived from CD8 to the transmembrane domain region primarily derived from CD28. The intracellular domain evolved in multiple iterations from a first generation developed in 1993 with CD3ζ, DAP12, or DAP10. This first generation proved not to be very effective in the clinic. By 1998, a significant leap was achieved in the second generation by adding costimulatory domains such as CD28, introduced by the Sadelain group, and later 4-1BB and 2B4 for NK cells. This second generation showed persistence and utility in the clinic. The third generation built on adding multiple costimulatory domains, while the fourth added the cytokine component or cytokine receptors and STAT binding domains. Therapeutic antibodies can directly mediate ADCC by NK cells expressing CD16. Immune checkpoint blockade directed against PD-1, CTLA-4, and TIGIT is another strategy that removes the brakes on NK cells. Bi-specific engagers mainly target CD16 and link it with tumor antigens, while tri-specific links CD16 and an activating receptor on NK cells to an antigen on tumor cells. Another modality utilizes an antigen recognition domain that binds to inhibitory ligands coupled to intracellular domains of activating receptors. This strategy converts inhibition of the TME into an activation signal. Seven levels of intervention to enhance anti-tumor activity: 1- Improving collaboration between NK cells and other effector cells such as T-cells, DCs, Macrophages, and Neutrophils. 2-armoring NK cells with cytokine signaling, 3- Engineering NK cells to improve persistence, metabolism, and resistance to exhaustion, 4- Preventing immunosuppression by TGFβ1, IDO, PGE2, adenosine, 5- Improving NK cells homing into tumors, 6- Preventing tumor angiogenesis and immunosuppression, 7- Reducing immunosuppression by MDSCs, T-regs, and TAFs

TGFβ is one of the main driving forces in the TME to exhaust NK cells. It suppresses NK cells by the induction of miR-183, which binds and represses DAP12 transcription/translation, a common dysfunction in NK cells infiltrating lung cancers.^505^ TGFβ helps cancer immune evasion by converting NK cells into exhausted type1 innate lymphoid cells with reduced anticancer activity (Fig. 8) and sequestration in tissues due to overexpression of α1 integrin and CD103.^183^ NK cells exposed to TGFβ or to its relative Activin, acquire a gene signature and phenotype similar to the less cytotoxic ILCs, becoming unable to control tumor growth in mice,^183–185^ suggesting a possible mechanism of NK cells exhaustion by reverting to an ILCs state. Engineering efforts that effectively addressed this issue were the introduction of a dominant negative of the TGFβ1 receptor, which competes with the endogenous TGFβ1R^506^ and separately, in another study through the knockout of TGFβR2.^507^

Knockdown of IDO in cancer cells increased their sensitivity to NK cells in vitro and promoted their accumulation in the tumors in vivo.^458^ L-Kynuenie, generated through IDO, depletes tryptophan, starves immune cells, impairs NK cell viability, and inhibits the upregulation of NKp46 and NKG2D under IL-2 stimulation. IDO is upregulated in cancer cells, APCs, and endothelial cells by TGFβ, IFN-γ, PGE2, PD-1, CTLA-4, IL-6, and TNF-α (reviewed in ref. ^508^). Therefore, IDO inhibition must be coupled with other modalities, such as immune checkpoints inhibitor of PD-1/PDL-1.

PGE2 and IDO suppress NK cells.^448^ Specifically, PGE2 increases T-reg cell activity in lung cancer by signaling through PGE2 receptors, with receptor EP4 being the most potent in further antagonizing NK cells. Similarly, PGE2 impairs the NK cell and DCs interactions, reducing IL-12 secretion by DCs and CD4 T-cell polarization. Tumor PGE2 was also reported to inhibit chemokine receptors on cDC cells. Most importantly, PGE2 could induce PD-L1 expression.^509^ Therefore, PGE2 inhibition must be also coupled with other modalities, such as immune checkpoints PD-1/PDL-1. Inhibitors of the Cox2-PGE2 axis, such as Celecoxib, cause bleeding and cannot be used long-term. Therefore, targeting the EP4 receptor with antagonists in combination with PD-1/PDL-1 would be more efficacious (reviewed in ref. ^510^).

Extracellular ATP rises in pathological conditions such as inflammation, ischemia, tumorigenesis, and hypoxia. In tumors, this extracellular ATP is converted into immunosuppressive adenosine using two ectonucleotidases CD39 and CD73 expressed on cancer cells, T-cells, T-regs, macrophages, neutrophils, MDSCs, NK, and the vasculature, thereby enriching the TME with high levels of adenosine^511^ (Fig. 8). Adenosine binds to widely expressed adenosine receptor A2AR, including on NK cells, macrophages, and DCs. The immunosuppression of adenosine is illustrated by tumor rejection in more than 60% of A2AR -deficient mice^407^ and promoting the accumulation of highly cytotoxic CD56^dim^ NK cells with upregulation of CX3CR1 transcription in NK cells, suggesting that adenosine prevents NK cells maturation and infiltration into tumors.^512^ Further, emphasizing the impact of adenosine inhibition of NK cells is the synergy between anti-PD1 and A2AR inhibitors, which inhibited metastatic melanoma and was primarily dependent on NK cells and IFNγ more than CD8^+^ T-cells.^513^ Blocking ATP hydrolysis using antibodies against CD39 and CD73 prevented adenosine accumulation, stimulated DCs and macrophages, and restored T-cell anti-tumor activity.^514^ Therefore, reducing adenosine accumulation in combination with ICIs is an efficacious strategy.

In another mechanism of tumor resistance to NK cells, tumor cells up-regulate collagen expression to enhance adhesive structures in the TME. This consolidation of collagen protects tumor cells by binding to soluble leukocyte-associated Ig-like receptor-1 (LAIR-1) expressing NK cells. Upon engagement with tumor collagen, human LAIR-1 associates with SHP-1 and SHP-2 and proceeds to dephosphorylate VAV1, thereby dampening NK activation (Fig. 6).

### Restoring dysfunctional NK cells

The impact of immune checkpoints on lifting exhaustion in T-cells is well established. However, for NK cells, the state of exhaustion is not well defined, as NK cells require at least two activating signals in addition to cytokines to mature and operate. These conditions are challenging to produce in the TME, where NK cells are usually dysfunctional.^515–518^ However, even NK cells that are being created in the bone marrow could be affected by the growth of remote tumors in mice by a process involving the downregulation of IL-15Rα+ cells among bone marrow stromal cells and the interrupted maturation of NK cells,^519^ a dysfunction that is remedied by IL-15 administration. Immature NK cells found in tumors were also reported in humans, and their presence was correlated with poor survival.^520^ Overall, an NK cell could be rendered dysfunctional remotely, even before it encounters a cancer cell. A transcriptional profile for these dysfunctional/exhausted NK cells is the low expression of T-Bet and Eomes transcription factors^521^ necessary to sustain maturation, identity, and anticancer activity,^522^ as well as CD16 and KIRs expression.^153^ In the early TME, Cb11+ myeloid cells (Basophils, monocytes, macrophages, and DCs), express soluble IL-15, and this contributes to the inflammatory response that helps NK cells proliferate in the early tumor stage, making IL-15, an activating component of the early TME, but later, due to mounting immunosuppressive forces in established tumors, IL-15 production diminishes.^523^ Additionally, NK cells that engage cancer cells and interact with other cells in the TME could be exhausted due to an overwhelming multitude of immunosuppressive factors and lack of activating cytokines with increased expression of inhibitory receptors including, NKG2A, CD96, PD-1, and TIGIT,^524–528^ as well as an across the board downregulation of major activating receptors which include DNAM-1, NCRs, NKG2D, CD16.^282,529–531^ The function of NK cells in cancer patients could be restored by chemotherapy,^532–534^ through multiple mechanisms, or by surgical removal of the primary tumor.^535^ NK cell function will most likely also depends on the tumor burden. Therefore, these modalities may be necessary to complement new and emerging clinical interventions.

### Immune checkpoints blockades

Since NK cell response integrates both inhibitory and activation signals, the blockade of any inhibitory receptor should enhance NK cell activation. Using ICI in the form of antibodies that bind these receptors and block the binding of their cognate ligands from cancer cells has shown a clear impact in T-cell immunotherapies, especially for PD-1 and CTLA-4. The participation of NK cells alongside T-cells in these therapies was noticeable,^525,536–538^ especially in MHC I-defective tumors. However, recent studies showed minimal PD-1 expression in NK cells from tumors, raising questions about its importance in PDL-1-expressing tumors.^539^ The same study suggested that TIGIT is markedly upregulated in these NK cells. CTLA-4 blockade with Ipilumab’s impact on NK cells is not clear. However, it was found to operate through the elimination of T-regs by NK cells mediated ADCC.^540^ CTLA4 engagement with ligands leads to its phosphorylation and recruitment of SHP-1 and SHP-2, leading to VAV1 dephosphorylation. After engagement with PDL-1, PD-1 ITSM domain phosphorylation at Tyrosine Y248 recruits SHP-2, suppressing NK cell activation (Fig. 6).

TIGIT is consistently upregulated in NK cells in human primary tumors and viral infection.^539^ TIGIT blockade reverses the exhaustion of NK cells from colon cancer patients and promotes their antitumor responses in mouse models.^524^ Additionally, NK cells expressing low TIGIT are resistant to MDSCs inhibition,^432^ suggesting the importance of this receptor in the crosstalk within the TME. In patients with metastatic melanoma, functionally impaired/exhausted, NK cells upregulated TIM-3 in NK cells compared to healthy subjects, and TIM-3 blockade in vitro reversed this exhausted phenotype.^541^ In T-cells, TIM-3 engagement with ligands leads to the phosphorylation of two tyrosines in its cytoplasmic tail (Y256 and Y263), leading to the dissociation of HLA-B associated transcript-3 (Bat-3). This dissociation disrupts LCK, ZAP70, and TCR activation.^542^ However, disengaged Bat-3 can also associate with P300, leading to transcription of MDM2, P21, BCL2, and the acetylation of P53, which may slow NK cell proliferation. Engagement of TIGIT with PVR and its phosphorylation through the Src- family kinases Fyn and Lck results in SHP-1 and SHP-2 recruitment, which in turn downregulates the PI3K, MAPK, and NF-KB signaling pathways and promotes VAV1 dephosphorylation. LAG-3 is highly expressed in T-cells from Hodgkin lymphoma and leukemia patients, and its synergy with anti-PD-1 was evident.^543^ In vitro, chronic stimulation of NK cells leads to epigenetic changes, upregulation of LAG-3 and PD-1, and NK cell dysfunction.^544^ LAG-3 mediated inhibition controls AKT phosphorylation and STAT5 activation leading to reduced mitochondria mass and quiescence.^545^ However, LAG-3 impact on NK cells remains obscure and needs more investigation. This might be due to the presence of many inhibiting receptors on NK cell surface that oppose any activation by ICIs. Additionally, a significant obstacle to ICI success is the simultaneous co-expression of many ICs in T-cells and probably NK cells, causing ICI failure.^546^

### Eliminating NK cells fratricide

Acquisition of tumor antigens by NK cells through engagement with cancer cells in a membrane transfer process called trogocytosis could lead to the misidentification of these NK cells as targets and their death by other NK cells. Interaction between ligands and receptors leads to the loss of the membrane patch harboring the ligand to NK cells. The example of NKG2D interaction with ligand RaeI revealed that this process required clathrin-dependent internalization of NKG2D, leaving RaeI on the cell surface for a period of at least 24 h.^547^ RaeI-dressed NK cells do not kill each other, suggesting they lost NKG2D in this process and are only killed by cells that did not interact with cancer cells. The loss of NKG2D would render these trogocytic NK cells anergic. In mice, the interaction of receptor 2B4 on one NK cell with its ligand CD48 on another NK cell was reported to prevent fratricide. Blocking this interaction with antibodies led to fratricide.^548^ Because CD48 is expressed on all nucleated hematopoietic cells, it may provide a non-MHC mechanism of self-tolerance. Fratricide is worsened when using ADCC and CAR-NK mediated therapies that will increase the visibility of NK cells if they express the antigen naturally or acquire it by trogocytosis. Multiple myeloma expressing high levels of CD38 and targeted with anti-CD38 Daratumumab (Dara), reduces NK cell number due to ADCC-mediated fratricide.^549^ Knockout of CD38 in expanded primary NK cells prevented Dara-induced fratricide in NSG mice, which are devoid of NK cells.^550^ However, host NK cells would probably be targeted by ADCC if the patient is not lymphodepleted first. A similar strategy using iPSCs cells FT576, depleted of CD38 in combination with Dara, prevented fratricide and showed efficacy in preclinical models, opening a path to clinical translation.^551^

However, the fratricide caused by trogocytosis cannot be eliminated by knockout of the antigen on NK cells. Rezvani et al. cleverly put a brake on killing trogocytic NK cells by adding a self-inhibitory iCAR targeting co-receptor Cs1 which is expressed on all NK cells and transmits an inhibitory signal via the KIR2DL1 cytoplasmic domain in iCAR. CD19^+^ cells did not express Cs1 and were targeted by an additional CAR against CD19.^552^

### NK cell engagers

Contact between immune cells and cancer cells can be encouraged by “engagers” that serve as a bridge by binding simultaneously to an activating receptor on the immune cell and a tumor-specific antigen on the cancer cell. Bite, trike, or tetraspecific engagers involve 2 or 3 or 4 receptors to strengthen the engagement and increase activation signals. The attractive aspect of engagers is the non-need to genetically modify immune cells and primarily target host immune cells to reactivate them. Early generations of engagers designed for T-cells and targeting CD3 have shown some success in hematological disease, with some toxicities that limited their efficacy. Among them, Blinatumomab, the first bite approved by the FDA, is a dual CD19 and CD3 engager. Blinatumomab is used to manage minimal residual disease after chemotherapy but is ineffective for certain patients who may relapse due to loss of CD19 or T-cell exhaustion. Several toxicities were reported for Blinatumomab, including neutropenia, neurotoxicity, infection, and cytokine release syndrome. Indeed, CD3-targeting engagers have been associated with severe toxicity, as in the case of Duvortuxizumab (anti-TAA+anti-CD3) and AFM11(anti-CD16a+anti-CD3). New generations of engagers seem to shift focus on the potential of innate immunity and enhancing its role in helping adaptive immunity. Three trifunctional NK cell engagers, targeting NKp46 and CD16 on NK cells and a tumor antigen on cancer cells (CD19, CD20, and EGFR) were shown to enhance cancer cell killing by human primary NK cells in vitro and in mice models.^553^ Another NK cell engager, AFM13 (CD16 + CD30), used with cord blood NK cells, exhibited enhanced killing of CD30^+^ leukemia and lymphoma targets.^554^ Several clinical trials evaluate multiple engagers, mainly targeting T-cells,^555^ with one ongoing trial NCT04074746 (Table 1) evaluating CB NK cells combined with AFM13 against R/R CD30^+^ Hodgkin lymphoma and non-Hodgkin lymphoma (reviewed in ref. ^556^).

## Engineered NK cells

The therapeutic efficacy of non-engineered NK cells is suboptimal, and using autologous unmodified NK cells is not conducive to better antitumor activity.^470–472^ Similarly, autologous NK cells derived from cancer patients are particularly weak candidates as they may be already in exhausted and dysfunctional states^516,528^ that could even be beneficial to tumors.^373,374^ These states suggest NK cell’s plasticity and ability for re-education in the tumor microenvironment. Therefore, understanding how NK cells are co-opted in tumors may help design better strategies to engineer resilient and incorruptible NK cells.

### Clonal cell lines a canvas still waiting for art

The first NK-based clinical trial was done using the cell line NK-92, which was originally derived from a patient’s blood with diffuse lymphadenopathy, B-symptoms and circulating LGL. Despite aggressive chemotherapy the patient passed of progressive lymphoma, roughly five weeks after admission. For this reason, the FDA requires NK-92 cell irradiation prior to administration. NK-92 is an obligate IL-2-dependent cell line with an anti-tumor activity superior to other NK cell lines and has a high safety profile despite its allogenic nature.^465^ NK-92 genome is aneuploid with a heterozygous stop mutation in the P53 gene. Clonal NK-92 cells are CD56^bright^, CD16^neg^, and KIR^neg^, a phenotype close to the recently identified NK2 population,^42^ making them a plausible descendent of the ILCP lineage. Whether it is possible to reprogram/reeducate NK-92 into a clonal cell line similar to an NK1 or an NK3 subset, is an intriguing question. Similar clonal NK cell lines were isolated (Table 2), but NK-92 unique characteristics, such as lack of KIRs and ease of genetic engineering are not yet fully exploited. This is probably due to the modest results using NK-92 in a phase I clinical trial, which showed minor responses in two patients out of twelve^81^ and another using CD33-CARNK-92 which showed safety but obvious clinical efficacy.^557^ The reported lack of efficacy in another phase I clinical trial for refractory and relapsed acute myeloid leukemia^558^ was attributed to circulating exosomes carrying an immunosuppressive cargo and disabling NK-92.^559^ Another clinical trial reported safety and some evidence of efficacy.^560^ A significant impediment to NK-92 use is the requirement for its irradiation. Thirty-one years after its first isolation,^465^ and despite widespread use, no reports of spontaneous IL-2-independent NK-92 clones exist. NK-92 does not cause tumors in ICR/scid mice even when supplemented with exogenous IL-2 or producing its IL-2.^561,562^ The risk that NK-92 cells could proliferate in vivo without a sustaining signaling has not been demonstrated in vivo. Still, it is speculated based on anecdotal findings with different tumor cell lines that caused subcutaneous nodules when implanted in terminal cancer patients despite failing in healthy volunteers.^563^ Additionally, numerous studies have shown no association between blood transfusion from precancerous blood donors and non-Hodgkin lymphoma risk,^564–566^ suggesting the unlikelihood of allogenic transfer of cancerous cells in healthy recipients. However, it cannot be excluded that NK-92 cells could proliferate if driven by self-sustaining IL-2 stimulation in severely immunodeficient patients. Therefore, combinations of tumor-suppressing signaling and suicide switches such as prodrug activating cytochrome P450 and HSV-TK enzymes or drug-activated iCasp9 switch, all inserted in multiple and separate chromosomal locations in the NK-92 cells genome could be a convincing step toward the potential use of this remarkable cell line in cancer patients, safely. This is possible through multiple rounds of infection and selection. However, more effort should be first deployed to improve the clonal cell lines’ efficacy by first determining the optimal signaling that drives cytotoxicity and resistance to exhaustion, followed by deciding what series of genes to add to improve tumor homing and strengthen activation signaling and what genes to eliminate, including ICs, to reinforce all these aspects and further enhance safety. Unfortunately, groups improving NK cell lines are rarely funded. Since its isolation, NK-92 and many other human NK cell lines have been instrumental in elucidating the biology of NK cells. However, except for NK-92 none of them advanced to clinical use in humans. The translational importance of NK cell lines will probably be more evident if they were to be used to treat animal cancers. A canine NK cell line (CN89)^567^ was reported as CD5^+^, CD8^+^, CD45^+^, CD56^+^, CD79a^+^ and NKp46^+^. However, its IL-2-independence and the B-cell marker CD79a^+^ as well as the absence of reports of cytolytic activity, cast doubt about its antitumor activity. While many canine clinical trials are ongoing (reviewed^568^), no canine NK cell line is being tested. However, the possibility of using human NK-92 for canine cancer by blocking xenoreaction with immunosuppressors is suggested by a phase I clinical trial in dogs, where a human T-cell line derived from child leukemia called TALL-104 was used safely.^569^ Unlike other T-cells, TALL-104 has lost its MHC I dependence and become an MHC I non-restricted T-cell line, much like NK cells. No toxicity to dogs was observed in the clinical trial. Seven dogs out of 19 showed a response, with one complete remission. Cyclosporin, an immunosuppressor was administered to dogs prior to TALL-104 infusion to prevent an anaphylactic reaction.

**Table 2:** List of human clonal NK cell lines

Cell line	Year established/published	Patient diagnosis	Age of donor	Sex	EBV status	Cytokine dependence	Clinical Trials
NK3.3	1982	Cloning of primary MLC-activated cells in a medium containing interleukin-2	N/A	N/A	EBV−	IL-2-dependent	NO
YT	1983	Acute lymphoblastic lymphoma (with thymoma)	15	Male	EBV+	IL-2-independent	NO
NK-92	1992/1994	LGL-NHL	50	Male	EBV−	IL-2-dependent	Yes (Table 1)
NKL	1996	NK-LGLL	63	Male	EBV−	IL-2-dependent	NO
NK-YS	1996	NK cell lymphoma, Nasal angiocentric, Leukemic state with systemic skin infiltration	19	Female	EBV+	IL-2-dependent	NO
KHYG-1	1997	Aggressive NK leukemia	45	Female	EBV−	IL-2-dependent	NO
HANK1	1998	Nasal-like NK/T-cell lymphoma	46	Female	EBV+	IL-2-dependent	NO
SNK-6	1998	Nasal NK/T-cell lymphoma	62	Male	EBV+	IL-2-dependent	NO
SNT-8	1998	Nasal NK/T-cell lymphoma	48	Female	EBV+	IL-2-dependent	NO
IMC-1	2004	Aggressive NK cell leukemia	42	Male	EBV−	IL-2-dependent	NO

*LGL* large granular lymphocyte, *LGLL* large granular lymphocyte leukemia, *NHL* non-Hodgkin’s lymphoma, *EBV* Epstein-Barr virus

### Chimeric antigen receptors (CARs) advances in the clinic

CARs are synthetic constructs emulating the TCR function but without the HLA requirement developed first for T-cells (CAR-T). They comprised in their first iteration (first generation) an extracellular antigen recognition domain, which is the single-chain fragment variable (ScFv) derived from an antibody tethered to a transmembrane domain and the intracellular activation domain CD3ζ chain (Fig. 8). The binding of ScFv to a specific antigen triggers activation. However, this design allowed very short-term proliferation. The second-generation CARs added the CD28 activation domain, later reinforced by another costimulatory molecule, CD134 (OX40), or CD137 (4-1BB) for CAR-T^570^ and 2B4^571^ or CD137 for CAR-NK.^572^ The latest and fourth generation added cytokines IL-12 expression under the control of the NFAT_6_ minimal promoter that initiates IL-12 transcription upon CAR-T-cell activation.^573^ In another approach, cytokines such as IL-15 could be produced constitutively (reviewed in ref. ^574^).

Novel strategies to convert inhibition into activation are emerging. For example, TGFβ immunosuppression in the TME can be converted into activation by tethering TGFβR2 extracellular domain to NKG2D cytoplasmic domain.^575^ Another strategy converted IL-4 suppressive signals in the TME into proliferative signals from the ectodomain of the IL-4 receptor to the cytoplasmic domain of the IL-7 receptor.^576^ NKG2D, a specific NK activator and specifically its extracellular recognition domain, could replace the ScFv and be tethered to DAP10 and CD3ζ.^577^ These innovative strategies seem to work with NK cells and may improve immunotherapies. The mere expression of CAR in NK cells may enhance the baseline signaling through the interaction of endogenous cell components with the costimulatory domains, even without antigen stimulation. This phenomenon has been reported for CAR-T and is termed tonic signaling and has been explained by the heterodimerization of CAR’s CD28 with the endogenous CD28.^578^ We have seen a comparable effect in NK-92 expressing CD28-based CAR, leading to faster cell growth than naïve NK-92, without engagement with antigen (Chen et al. unpublished data). In T-cells, CARs induce cytotoxicity and proliferation by producing an autocrine loop of cytokines, whereas in NK cells they only induce cytotoxicity. Therefore, in contrast to CAR-T, CAR-NK cells need the addition of cytokines for survival and metabolic fitness.

Four representative clinical trials using different sources of NK cells (cord blood, iPSCs, and PB NK) are discussed here. Rezvani et al. in a clinical trial initiated in 2017 (NCT03056339), reported in 2020 phase I interim results.^90^ These showed that cord blood CAR-NK-CD19 cells armored with soluble IL-15 could persist in patients for over a year with a single infusion, with an overall response rate (ORR) of 73% and achieving complete remission (CR) for seven out of eleven patients, without any cytokine related syndrome (CRS), graft versus host disease (GVHD), Immune effector cell-associated neurotoxicity syndrome (ICANS) or NK-related toxicity as. The phase1/2 results were recently reported,^579^ with an ORR (d30)= 48.6%, ORR (d100)= 48.6%, 1 year: Overall survival (OS) = 86%, and progression-free survival (PFS) = 32%, with no CRS/ICANS/GVHD. Notably, the patients who achieved higher ORR had higher levels and longer persistence of CAR-NK cells. Most of these patients received a lymphodepleting nonmyeloablative preparative regimen of cyclophosphamide and fludarabine prior to CAR-NK infusion and received follow-up treatment 30 days post-infusion. Studies have shown that lymphodepletion enhances CAR-T efficacy by eliminating “cytokine sinks” competition by T-regs and other competing immune system elements.^580^ Lymphodepletion conditions the immune system to eliminate regulatory mechanisms that could hinder the functioning of infused CAR-T cells. Therefore, lymphodepletion prior to infusion most probably enhances CAR-NK efficacy. Furthermore, radiation treatment induced CXCL8 secretion from tumor cells and enhanced the directional migration of CD56^dim^ NK cells to the tumor.^581^ Conditioning and multifactorial therapies will be even more necessary to give cell immunotherapies a winning chance against the more challenging solid tumors.

So far, the US Food and Drug Administration (FDA) has only approved CAR-T cell therapies against hematological cancers. Solid tumor heterogeneity and antigenic diversity, in addition to the TME immunosuppressive factors, are challenging. Therefore, better design of CARs incorporating other activation signals such as STAT3 and STAT5^582^ and other useful activators that may allow better survival of CAR-NK cells in the TME if combined with other modalities.

Another clinical trial (NCT04245722),^583^ used FT596, an off-the-shelf, CAR-NK-CD19 cell therapy derived from iPSCs, in patients with relapsed/refractory (R/R) B-cell lymphomas (BCLs) and chronic lymphocytic leukemia. FT596 employs three anti-tumor strategies: (1) a proprietary CD19-targeting CAR; (2) a high-affinity, non-cleavable CD16 Fc receptor that facilitates tumor targeting and enhances ADCC when paired with a therapeutic mAb; and (3) an IL-15/IL-15 receptor fusion that promotes cytokine-autonomous persistence. Preclinical in vivo models of leukemia and lymphoma have shown FT596 CAR-mediated effectiveness against CD19^+^ tumor cells. Additionally, when combined with the anti-CD20 agent rituximab, FT596 was effective against both CD19^+^ and CD19^−^ tumor cells.

In the interim results reported in 2021, No dose-limiting toxicities, no ICANS/GVHD, but two cases of CRS were reported. After the first FT596 treatment cycle, ORR was observed in 5/8 patients receiving FT596 as monotherapy; when combined with Rituximab the ORR was obtained in 5/9 patients. At single-dose levels of at least 90 million FT596 cells as monotherapy, 8 of 11 patients achieved an OR, including 7 CRs. Among the 4 patients with prior CAR-T cell therapy treatment, a dose of at least 90 million FT596 cells, achieved CR in two patients. Most importantly, No B- or T-cell mediated anti-FT596 responses were seen. These results demonstrate the efficacy and safety of off-the-shelf NK cells derived from iPSCs. Noteworthy is the use of conditioning chemotherapy (cyclophosphamide and Fludarabine) prior to cell infusion.

In clinical trial NCT03415100, Xiao et al.^584^ used a novel chimeric antigen receptor (CAR) combining the extracellular domain of the natural killer (NK) cell receptor NKG2D with DAP12. Expression of the NKG2D-RNA-CAR significantly enhanced NK cell cytolytic activity in vitro, and in vivo in mice. The clinical trial interim results reported in 2019 showed that three patients with metastatic colorectal cancer were treated with local infusion of CAR-NK cells. Two patients experienced reduced ascites generation and a marked decrease in tumor cells in ascites samples (2/2, RECIST: SD), while one patient exhibited rapid tumor regression and a complete metabolic response in treated liver lesions (1/1, RECIST: SD). This small sample clinical trial shows the potency of PB NK cells when activated with hybrid chimeric antigen receptors based on NK cell receptors.

Another phase 1 clinical trial (NCT06325748) is currently enrolling adult patients with R/R CD33 and/or FLT3 expressing heme malignancies for allogenic treatment using SENTI-202, a Logic Gated off-the-shelf CAR-NK cell therapy candidate that selectively targets hematologic malignancies, using three technologies: 1) the OR GATE, which is an activating CAR that targets either or both CD33 and FLT3, 2) the NOT GATE, which recognize and protect healthy cells from being killed. And 3) a calibrated release of IL-15 to increase the persistence/expansion and activity of CAR-NK cells and potentially the host immune cells. This first-in-man trial will inform on the potential of this novel technology.

### Engineered NK cells and the persistence problem

Infused allogenic haploidentical NK cells do not persist for more than 3 weeks and are eliminated by the recipient patient’s immune system.^89,471,585,586^ However, this rejection could be delayed by lymphodepletion,^79^ which, although not required in autologous NK cell transfer might improve NK cytolytic activity as mentioned earlier and especially when T-cells are weakened in the recipient.^587^ The most used lymphodepleting agents, cyclophosphamide or Fludarabine are given for one week. Lymphodepletion also eliminates “cytokine sinks” competition by T-regs and other competing immune system elements.^580^ The importance of lymphodepletion for NK cell expansion in the recipient is clearly demonstrated by the increased number of NK cells settling in the bone marrow when lymphodepletion intensity increases.^588^ Autologous NK cells would, in theory, persist more if the conditions that incapacitated/exhausted them in the cancer patient were removed. For this reason, we believe that engineering autologous NK cells, for example, by depletion of inhibiting receptors and increasing activating signaling, is a logical approach. Knockdown of HLA in haploidentical (half-identical) NK cells, assuming it is easily doable and does not lead to fratricide through missing self, may increase persistence and delay elimination by the recipient immune system. This approach was shown to work for human T-cells.^589^ A more practical solution is increasing the intensity of lymphodepletion, which may be an effective way to increase persistence, especially when combined with cytokine production by infused NK cells.^590^ Another possible therapeutic option could be the adaptive NK cells NKG2C^high^CD57^+^ that expand in humans infected with HCMV.^179,180^ Interestingly, these cells downregulate PLZF, making them probably less susceptible to reverting to a less cytolytic ILC1 phenotype. These adaptive NK cells have significant persistence,^591^ pronounced ADCC, resistance to MDSCs and are intrinsically resistant to Treg cells.^592^

### TCR-dressed NK cells

Engineering TCRs in allogeneic T-cells is a significant challenge since introduced TCRs will form mispaired non-specific TCRs.^593,594^ Therefore, expressing TCRs in NK cells that lack TCRs is a more sensible approach.^595^ TCRs that recognize tumor antigens presented by MHC can bind to all cellular antigens, including intracellular antigens unreachable by CAR-T and CAR-NK. CARs target only membrane proteins, which are encoded by one-fourth of the human genome,^596^ leaving 75% of proteins out of reach. This reduces the usefulness of the CARs approach, which also suffers from tumor antigen escape.^597^ Additionally, targeting normal overexpressed antigens by CARs can veer off-target or completely deplete normal tissue. This approach is exemplified by the anti-CD19 CAR strategy, which kills all B-cells,^598^ leaving cured survivors with a permanent need for antibody infusion and a lack of response to vaccination in pandemics.^599^ Tumor-infiltrating T-cells isolated from tumors often express tumor-specific TCRs.^600–602^ However, they may already be exhausted.^603,604^ NK cells naturally attack tumors in an MHC-independent manner, clearing tumors that antigen-specific T-cells cannot possibly target.^38,605,606^ NK cells are activated when MHC I expression is downregulated in transformed^607^ and virus-infected cells.^170^ The acquisition of resistance phenotype by tumor cells is often caused by the expression of inhibitory signals from MHC I.^608^ Indeed, HLA-E and HLA-G inhibit tumor cell lysis by NK cells.^608,609^ Therefore, combining the TCR-antigen-MHC-dependent recognition with innate MHC-independent tumor recognition will expand NK cells’ killing repertoire. TCR activation enables T-cells to manufacture IL-21 in an autocrine loop^610^ to activate Stat1^611^ and Stat3.^612^ Stat3 enhances telomeres maintenance.^613^ TCR activation also allows the production of IL-2,^611^ which promotes T-cell proliferation by activating STAT5. Finally, TCR activation prolongs cytokine signaling by downregulating CIS and Socs3.^614^ However, since CAR-NK and TCR-NK cells do not proliferate in response to antigens, as they require cytokines, adding a potent cytokine such as IL-15, IL-21, IL-2, or their combination will enhance the longevity and fitness of NK cells. The persistence of NK cells is another pressing problem, and lessons learned from CAR-T show that the shortening of telomeres depends on patients’ age and that the loss continues during the manufacturing process of CAR-T cells. Indeed, introducing hTERT mRNA in CD19-CAR-T led to more persistence in vivo,^615^ and the exhaustion of CAR-T directed against melanoma correlated with telomeres length.^616^ Similar strategies are probably needed to enhance NK cell persistence. Two clinical trials are currently testing TCR-NK. NCT06383572, a phase I/II trial evaluating the safety, and effectiveness of PRAME-T cell receptor-natural killer (PRAME-TCR-NK) cells against AML, MDS, and relapsed/refractory multiple myeloma. NCT06083883 is another phase 1/1b trial evaluating an Affinity-enhanced T-cell Receptor (TCR) Against the NY-ESO-1 in patients with advanced synovial sarcoma and myxoid/round cell liposarcoma. Both clinical trials use lymphodepleting chemotherapy.

## Environmental factors

**Fig. 9:**
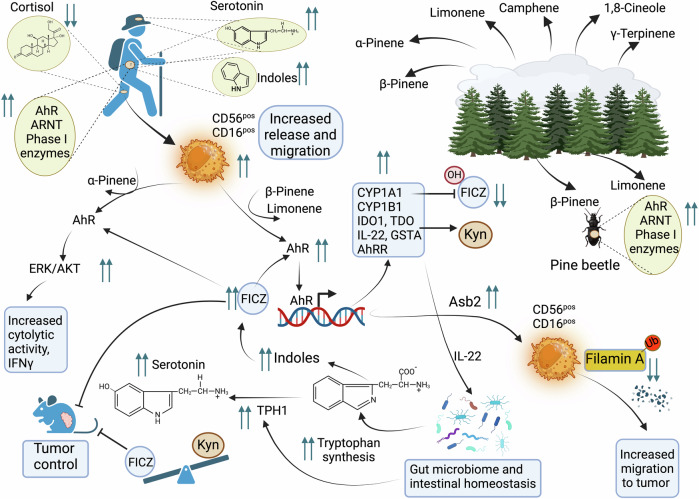
Impact of the environment on NK cells: Phytoncide model. From the pine beetle to humans, a response to xenobiotics via the xenobiotic response element is mediated by the Aryl hydrocarbon receptor, which is induced by natural compounds and environmental pollutants that bind and activate AhR. Endogenous compound FICZ derived from Tryptophan metabolism can bind AhR with high affinity and trigger the expression of phase I enzymes, IDO, TDO, and IL-22 with wide-ranging physiological effects. AhR activation by FICZ affects NK cytolytic activity, and migration to tumors, with possibly a shift in Tryptophan metabolism by the microbiome from the Kynurenine pathway, which mainly degrades tryptophan in the liver by TDO, and IDO, to the beneficial 5-HT pathway by TPH, in the gut and brain and the Indole pathway catalyzed by the gut microbiota to generate indoles such FICZ which reinforces this signaling loop and enhances cancer control

A series of studies evaluating the impact of the environment on human NK cells revealed that CD16^pos^ NK cell cytolytic activity could be increased in males and females to last up to 7 days by a simple walk in the forest “forest bathing” while a walk in the city did not,^617–620^ with, nevertheless, a surprising decrease of CD4 cells. More recent studies confirmed the increase in CD56^pos^ NK cells in a forest bathing group compared to an urban group.^621,622^ The earlier pioneering studies suspected phytoncides released from pine trees, which were confirmed to be the main factor in NK cell enhancement^623^ (Fig. 9). The relaxed feeling in the forest caused a decrease in the concentrations of cortisol in the blood and adrenaline in urine, suggesting the possibility of less immune inhibition from cortisol.^619^ A phytoncide (α-pinene) was shown to activate the ERK/AKT pathway in NK-92 cells and to increase their cytolytic activity,^624^ suggesting a direct effect on NK cells. Similar effects were noted for other phytochemicals, such as cymene and camphor. However, we hypothesize that the complex composition of phytoncide, which includes α-pinene, β-pinene, 1,8-Cineole, γ-Terpinene, Camphene, and Limonene, could have multiple targets that enhance innate immunity in particular NK cells. The Aryl Hydrocarbon Receptor (AhR) and aryl hydrocarbon receptor nuclear translocator (ARNT) are heterodimerizing transcription factors involved in sensing and responding to toxic xenobiotic chemicals by activating the transcription of CYP1A1, CYP1B1, IDO1, TDO, IL-22, GSTA and Aryl-Hydrocarbon Receptor Repressor AhRR. Recent work showed that in the pine beetle (*Dendroctonus armandi*), both AhR and ARNT were substantially induced by β-pinene and Limonene,^625^ leading to the induction of several phase I enzymes. IL-22 enhances Tryptophan synthesis in the gut and increases Trp hydroxylase (THP1), leading to Serotonin and endogenous tryptophan derivative, 6-formylindolo[3,2-b] carbazole (FICZ) production by deamination of Tryptamine.^626^ FICZ is also a potent ligand for AhR and can potentiate NK cell IFNγ production and cytolytic activity and control of tumors.^627^

More evidence is mounting to support AhR/ARNT role in mediating the transcription of genes involved in inflammation and control of the differentiation and activity of adaptive and innate immune cells.^628^ Additionally, NK cells stimulated by cytokine IL-2, IL-15, or IL-12 induce AhR expression, which can be activated by tryptophan derivative FICZ, the most potent ligand for the AhR produced endogenously and by gut microorganisms.^629^ Activated AhR also enhances the activation of the AKT serine/threonine kinase AKT pathway to promote cell survival.^630^ Activated AhR regulates NK cell migration through the Asb2 gene, which mediates the degradation of Filamin A via ubiquitination, leading to increased NK cell migration into tumors^631^ (Fig. 9). Therefore, we hypothesize that “forest bathing” might activate ERK/AKT by specific products such as α-pinene and induce AhR through β-pinene and Limonene. AhR is then activated by endogenous ligands, among which is tryptophan derivative FICZ, whose production by gut microbiota is promoted by phytoncide-AhR-induced IL-22. Interestingly, immunosuppressive tryptophan derivative Kynurenine, produced by IDO in tumors, can also activate AhR in T-cells to generate T-regs,^632^ inhibit CD4 and CD8 T cells,^456^ and reduce NK cytotoxicity.^457^ Therefore, tryptophan derivative FICZ and tryptophan metabolite Kynurenine have different impacts on AhR activation. However, FICZ binds to AhR in the nanomolar range and outcompetes kynurenine and its metabolites. Another metabolite of tryptophan is serotonin, which incidentally increases during forest bathing,^633^ suggesting that taking a walk in the forest may cause a shift in Tryptophan metabolism from the kynurenine pathway, which mostly degrades Tryptophan in the liver by Trp-2,3-dioxygenase (TDO), indoleamine-2,3-dioxygenase (IDO), to the 5-HT pathway by TPH, which takes place in both the gut and brain and the Indole pathway catalyzed by the gut microbiota to generate indoles such FICZ. Interestingly, the kynurenine pathway seems to dominate in tumors mostly through IDO, and consequently, tumors are depleted of Tryptophan. This suggests that supplementing indoles such as FICZ with its strong affinity to AhR or strengthening microbiota production of indoles, possibly by forest phytoncides, might counterbalance the kynurenine pathway inhibition of NK cells in tumors.

## Concluding remarks

**Fig. 10:**
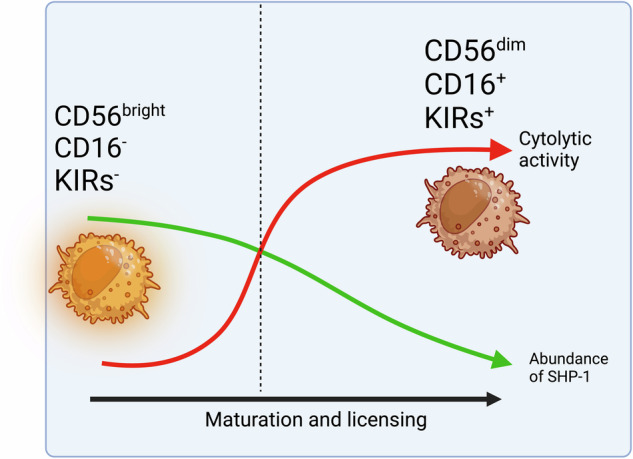
SHP-1 content is modulated by licensing and determines the degree of activation. CD56^bright^ uneducated NK cells respond to inhibitory signals with strong production of phosphatase SHP-1, leading to their rapid inactivation, while educated, licensed CD56^dim^ NK cells have reduced SHP-1 production when responding to inhibitory ligands, allowing them to resist deactivation

We attempted to review many aspects of NK biology in healthy and pathological conditions. How they are inhibited, and why their killing requires the synergy between multiple activation signals. NK cells can support angiogenesis in tumors and pregnancy and reduce T-cell overreaction to infection through IL-10 secretion to prevent tissue damage. They can also activate DCs, which in response, secrete IL-12 to enhance NK cell’s secretion of IFNγ, which is required for CD4 polarization. They help M0 and M2 macrophages transition to the pro-inflammatory M1 phenotype and induce monocyte conversion to DCs. They can detect and kill cancer cells lacking MHC I and senescent, stressed, and virus-infected cells. And can by delivering GNLY via nanotubes, specifically, kill bacteria in DCs, macrophages and trophoblasts, without harming host cells. We also examined the vast interactions with other immune cells of innate and adaptive immunity. It is clear at this point that NK cells are not created to do a unique specific task. This bewildering range of tasks will require many functionally adaptive or reprogramed NK cell states. This is probably achieved through origination from two lineages and egress at early stages from the bone marrow and subsequent wide variety of spatiotemporal education and maturation processes from which heterogenous NK cell populations emerge. Therefore, when using NK cells for therapeutic purposes, are these functionally adaptive/reprogrammed states of NK cells optimal? For example, the potential for reciprocal crosstalk between cytokine-producing CD56^bright^ subset, which accumulates in draining lymph nodes^634^ with comigrating neutrophils,^635^ suggests an important role of this subset in the development of adaptive immune responses. Similarly, the CD56^bright^, CD16^neg^ NK cell subset high antioxidative capacity and resistance to ROS produced by neutrophils^636^ suggests this subset more than the CD56^dim^, CD16^pos^ is more suitable to resist inhibition by tumor resident neutrophils and participate in the cross-talk between neutrophil NK and DCs. Additionally, the downregulation of NKp46 and NKG2D expression, induced by phagocytes produced ROS was observed in the CD56^dim^ but not the CD56^bright^ subset of NK cells.^637^ On the other hand, a clear advantage of CD56^dim^ and CD16^pos^ is their potential combination with therapeutic antibodies. Additionally, CD56^bright^, like uneducated NK cells respond to inhibitory signals with strong production of phosphatase SHP-1, leading to rapid inactivation (Fig. 10), while CD56^dim^, educated, licensed NK cells produce less SHP-1 when encountering these inhibitory ligands, allowing them to better resist inhibition.^145^

Similarly, clonal NK cell lines isolated from patients have distinct phenotypes reflecting NK cell diversity. They, however, provide a homogenous starting population that allows precise genetic engineering that is only limited by imagination. However, most genetic modifications on NK cell lines often implement progresses made in CAR-T for T-cells, not based on NK cell biology. For example, the NK-92 cell line, with its superior anticancer properties, has not been radically engineered to proliferate in vivo under suicide switches in animal models to test whether this proliferation can eradicate tumors. Studies showing that suicide switches can control NK-92 proliferation in vivo are also lacking. The fact that clinical trials using NK-92 showed little benefit suggests that NK-92 still needs further improvement by engineering. NK cells are among the early responders, and by lysing cancer cells, they expose tumor antigens to the care of DCs, which present them to T-cells in tumor-draining lymph nodes to induce polarization of CD4 T-cells into Th1 helper cells and the conversion of CD8 T-cells into cytotoxic T-cells (CTL). NK cells again intervene to allow the polarization of CD4 T-cells by secretion of IFNγ, which is also enhanced by IL-12 from DCs. This collaboration between DCs, NK, and T-cells is the prelude to establishing an effective adaptive anti-tumor immunity. The killing of immature DCs and activated T-cells by NK cells through the NKp30 and the modulation of MHC I HAL-E expression to prevent the killing or facilitate it, solidifies NK cell role in shaping adaptive immunity with significant consequences on CAR-T and NK-mediated immunotherapies. Therefore, strategies using NK cells will likely work if NK cells are engineered to enhance DCs and T-cell responses and to access and survive in the TME.

Immune checkpoint blockade has become a cornerstone in cancer immunotherapies. However, combinations with agents that block immunosuppression in the TME are necessary. Lymphodepletion and radiation therapy can help in this direction, but lessons learned suggest it will not be enough. An important direction is the genetic engineering of NK cells to create robust and versatile signaling incorporating several activation pathways found in NK cells without hyperactivating and exhausting NK cells. Another lesson learned is that NK cells can switch sides and play a supportive role in tumors and metastasis. Whether genetic engineering can preclude this is an important direction. Aside from cancer immunotherapy, NK cells can recognize stressed and senescent cells, and this developing area could prevent the development of cancers. This is an exciting direction for which genetically engineered NK cell lines might be more suitable.

CARs and TCRs not only enhance the killing of targeted cells but may also help the directional homing by collecting NK cells in antigen-rich tumors. Similarly, ectopic chemokine receptor expression could dramatically improve NK cells homing into tumors. Inhibition of IDO, PGE2, A2aR, and TGFβ, coupled with factors that enhance tumor perfusion, such as metronomic chemotherapy, which also increases NK cell recruitment into tumors, may enhance NK-mediated immunotherapies. Like CAR-T, the successes reported using CAR-NK immunotherapies are mostly for hematological cancers. Patients who respond can also relapse, suggesting either a lack of NK cells persistence, exhaustion, or possibly a conversion to a pro-tumorigenic function. While the two first possibilities could be remedied by genetic engineering, a re-education of NK cells in the cancer patient leading to a conversion into a less protumor phenotype would be a complex problem to solve. Such conversion could be determined by single-cell RNAseq analysis of CAR-NK cells from patients. For example, treatment with TFGβ1 converts NK cells to a less cytolytic state like ILC1s, presumably through the loss of EOMES. Blocking or reverting this conversion could restore NK cell cytolytic activity.

Another possible way to enhance NK function is by targeting intracellular checkpoints. VAV1 is the hub for various activating and inhibitory pathways, acting as a switch to turn off NK activation and prevent the downstream activation cascade^268^ (Fig. 5). Preventing VAV1 deactivation could offer a potent means to activate NK cells, with, however, the potential risk of higher toxicity to normal tissues. The dominant inhibitory signals originate from MHC I engagement with KIRs, leading to phosphorylation of their ITIMs domain, which creates a docking space for SHP-1 and SHP-2. The involvement of SHP-1 in VAV1, PIP3, and SLP76 dephosphorylation has been established,^265,638^ while SHP-2 dampens NK cells cytotoxicity, independently of KIR inhibition.^639^ Another regulator of NK cell function is CIS (cytokine-inducible SH2-containing protein). CIS is a member of the Socs family and targets STAT activation. The knockout of the CIS gene was reported to substantially improve CB-NK expressing soluble IL-15.^488^

The journey to enhance NK cell cytotoxicity and persistence is following in the footsteps of CAR-T development. However, there is a clear need to engineer activation signaling and co-stimulatory molecules specific to NK cells and their biology. The use of cytokine(s) and multiple activation signals will be mandatory. Incorporating elements to resist/modulate the TME will be necessary to allow NK cells to survive and engage in fruitful cross-talks with other effectors of immunity. The use of CARs, TCRs, ICI, engagers, radiation therapy, or bespoken chemotherapy regimens need to be optimized to increase the therapeutic efficacy of NK cells. All these improvements might benefit from immunomodulation by beneficial environmental factors and the strengthening of indoles-producing microbiota that enhance the immune response. These interventions could easily be incorporated into any treatment, and their beneficial impact merits further investigations.

## Forward-looking perspective

Since the early 1970s, substantial research efforts have aimed to unravel NK cells’ functions and killing mechanisms. This effort is dwarfed by the extensive focus on adaptive immunity cells discovered earlier and which are fundamental to vaccine efficacy. As our understanding deepens, it becomes evident that NK cells are diverse populations engaged in various roles, using a complex balance of activating and inhibitory receptor signaling. Unfortunately, this balance does not always lead to desired outcomes, such as in cancer patients.

Two recent groundbreaking studies have identified the progenitor of NK cells, emphasizing that EOMES is crucial in differentiating NK cells from non-cytolytic ILC1s. Interestingly, NK cells exposed to TGFβ or Activin acquire a gene signature similar to ILCs, suggesting that NK cell exhaustion might involve reverting to an ILC state. Another significant study revealed that NK cells could kill activated T-cells, including CAR-CD19 T-cells, a capability they already possess against immature DCs. This finding raises important questions: Do patient NK cells hinder current CAR-T therapies? Could NK-mediated immunotherapies undermine the patient’s T-cell compartment, limiting adaptive immunity? And might combining CAR-T and CAR-NK therapies be counterproductive? Using high-dimensional single-cell analysis of human natural killer cells, an equally groundbreaking advance delineated three major populations of mature NK cells in PB.^42^ Two populations, NK1 and NK3, originated from ENKPs, and another NK2, originated from ILCPs. Which of these populations is a perfect fit for natural killer therapies remains to be confirmed.

Promising advances in NK-mediated immunotherapies are emerging, with numerous clinical trials underway^90,487,590,640^ (Table 1), making the full potential of NK cells increasingly attainable. These advances are bolstered by novel signal transduction engineering and precise gene editing using CRISPR/Cas9 technologies. Single-cell RNA sequencing (scRNA-seq) offers a detailed view of individual NK cell transcriptomes, enabling the identification of distinct NK cell subpopulations and their functional states.^42^ AI-driven clustering algorithms can categorize these cells based on gene expression, identifying subsets with enhanced cytotoxicity or resistance to tumor-induced immunosuppression and uncovering key regulatory genes and pathways. AI can design next-generation CARs for NK cells based on specific NK activating receptors, avoiding issues of exhaustion and overactivation. AI can optimize the ex vivo expansion and activation of NK cells, enhancing their potency and viability. Personalized data can guide the design of genetically engineered NK cells tailored to individual patient needs. Additionally, AI can identify synergistic combinations of NK cell therapies with other treatments, such as checkpoint inhibitors or cytokines.

Engineering autologous NK cells could address persistence issues more effectively than haploidentical transfers, especially if the KIR advantage of allogenic NK cells could be replicated through gene editing. AI can also predict which haplotype would persist more in a given patient. CAR-NK therapies may offer a cost-effective alternative to current prohibitively expensive CAR-T therapies, potentially improving access to treatment. Banking NK cells for multiple uses in multiple patients, particularly for cells from CB, could help bridge this gap. However, more clinical trials in large animal models, such as dogs with spontaneous tumors, are needed to validate therapies for tumors with similar signatures in humans and dogs, like osteosarcoma. Given recent reports of CAR-T lymphoma risks,^641^ this approach could also test the safety of new immunotherapies due to potential risks associated with gene editing strategies, with possible off-target effects, which might also be seen in CAR-NK therapies if used at larger scales. In this regard, more research is needed to examine the safety of combining elements that could increase the fitness and survival of NK cells but may lead to a gain of function. NK cell immunotherapies are extending to autoimmune diseases such as SLE with several clinical trials launched and recruiting (Table 1). These are targeting the B-cell compartment for elimination via CAR-CD19-NK. For other autoimmune disorders, NK cells expressing the extracellular domain of PDL-1 can target autoreactive T cells, which overexpress PD-1. This strategy has shown efficacy in the preclinical model and might be applicable to autoreactive follicular T-cells.^642^ Another encouraging direction is the bourgeoning of NK cell engagers as a safe way to enhance NK cells without genetic modifications. However, it appears they will not be sufficient as monotherapy and must be combined with other modalities. Engineering engagers with cytokines such as IL-15 provide better survival to NK cells. (reviewed^41^).

This is a time of great promise. As we gain deeper insights into NK cell signaling and the molecular mechanisms of their activation and inhibition, advances in NK-mediated immunotherapies will accelerate, leading us toward a brighter future.
